# Tandem sulfofucolytic-sulfolactate sulfolyase pathway for catabolism of the rare sulfosugar sulfofucose

**DOI:** 10.1128/mbio.01840-25

**Published:** 2025-08-18

**Authors:** Adam W. E. Stewart, Jinling Li, Mihwa Lee, Jessica M. Lewis, Marion Herisse, Vinzenz Hofferek, Malcolm J. McConville, Sacha J. Pidot, Nichollas E. Scott, Spencer J. Williams

**Affiliations:** 1 School of Chemistry and Bio21 Molecular Science and Biotechnology Institute, University of Melbourne 200954, Parkville, Victoria, Australia; 2 Department of Microbiology and Immunology, University of Melbourne, at the Peter Doherty Institute for Infection and Immunity 534133 , Melbourne, Victoria, Australia; 3 Department of Biochemistry and Pharmacology, Bio21 Molecular Science and Biotechnology Institute, The University of Melbourne, 195116, Parkville, Victoria, Australia; Oregon State University, Corvallis, Oregon, USA

**Keywords:** organosulfur, metabolism, sulfoglycolysis, sulfur cycle, catabolism

## Abstract

The rare sulfosugar sulfofucose (6-deoxy-6-sulfo-D-galactose) is catabolized by *Paracoccus wurundjeri sp. nov*. strain Merri via a novel tandem pathway that couples sulfofucolytic and sulfolactate sulfolyase reactions. We isolated *P. wurundjeri* from soil and demonstrated its ability to utilize sulfofucose as the sole carbon source, leading to complete degradation of sulfofucose and release of sulfite. Proteomics and metabolomics analysis revealed a sulfofucolytic variant of the Entner–Doudoroff (ED) pathway, wherein sulfofucose is converted to sulfolactaldehyde through oxidation, lactone hydrolysis, dehydration, and retro-aldol steps. The sulfolactaldehyde is subsequently degraded to pyruvate and sulfite via a downstream biomineralization pathway involving sulfolactate as an intermediate. *In vitro* reconstitution of the key enzymatic steps validated the pathway, identifying four enzymes: SfcH (dehydrogenase), SfcD (lactonase), SfcF (dehydratase), and SfcE (aldolase), that collectively convert sulfofucose to sulfolactaldehyde and pyruvate. Our findings provide insight into microbial utilization of rare sulfosugars and expand the known metabolic versatility of the genus *Paracoccus*.

**IMPORTANCE:**

Sulfosugars, such as sulfoquinovose, are integral to the global sulfur cycle, but the metabolism of rarer sulfosugars, like sulfofucose, remains poorly understood. This study identifies a unique bacterial catabolic pathway for sulfofucose degradation, comprising a sulfofucolytic ED pathway, followed by biomineralization to sulfite. The identification of this tandem pathway highlights the metabolic flexibility of bacteria to adapt carbohydrate catabolic pathways for processing structurally similar sulfosugars. This work sheds light on the ecological role of sulfofucose metabolism and broadens our understanding of bacterial sulfur metabolism and biogeochemical sulfur cycling, and offers potential biotechnological applications.

## INTRODUCTION

Sulfur is an essential macronutrient and is incorporated into a wide range of organosulfur compounds that serve as metabolic currencies and facilitate sulfur exchange between producer and consumer organisms (1, 2). The biosynthesis and catabolism of these organosulfur metabolites form intricate biochemical networks within the global sulfur cycle (3, 4). Organosulfur compounds of global significance include the sulfur-containing amino acids cysteine and methionine, the osmoprotectants dimethylsulfoniopropionate (5) and dimethyloxosulfonium propionate (6), the short-chain organosulfonates 2,3-dihydroxypropanesulfonate (DHPS) and sulfolactate (SL) (7, 8), and plant and cyanobacterial sulfolipid (sulfoquinovosyl diglycerol; SQDG), which contains the sulfosugar sulfoquinovose (SQ, 6-deoxy-6-sulfo-D-glucose) (9).

**Fig 1:**
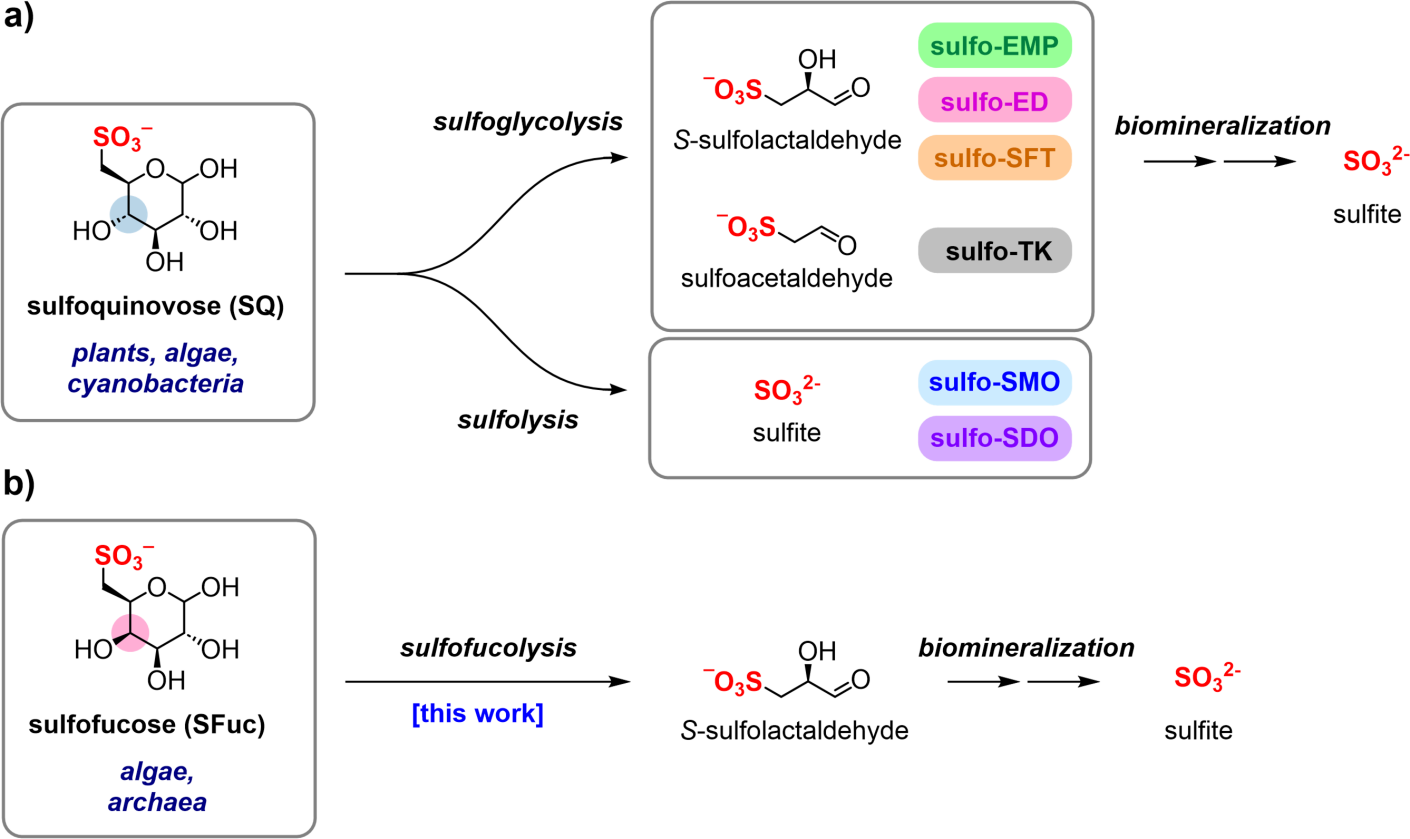
Breakdown of the sulfosugars sulfoquinovose and sulfofucose. (**a**) Degradation of sulfoquinovose to sulfoacetaldehyde (SA) or sulfolactaldehyde (SLA) by sulfoglycolysis (sulfoglycolytic Embden−Meyerhof−Parnas [sulfoEMP]; sulfoglycolytic Entner−Doudoroff [sulfo-ED]; sulfoglycolytic sulfofructose transaldolase [sulfo-SFT]; and sulfoglycolytic transketolase [sulfo-TK] pathways) or sulfolysis to sulfite (sulfolytic SQ monooxygenase [sulfo-SMO] and sulfolytic SQ dioxygenase [sulfo-SDO] pathways). (**b**) Sulfofucose has been identified in glycoconjugates from algae and archaea; this work discloses a two-phase sulfofucolysis−biomineralization breakdown pathway.

Over the past three decades, extensive work has elucidated the biosynthetic route to SQ in plants and cyanobacteria, as well as the diverse bacterial pathways used for its degradation (Fig. 1a) (9–12). Glycoside hydrolases known as sulfoquinovosidases initiate catabolism by cleaving SQ from glycoconjugates such as SQDG and sulfoquinovosyl glycerol (13–15). SQ breakdown then occurs via multiple sulfoglycolytic and sulfolytic pathways. In sulfoglycolytic bacteria, SQ is partially degraded into short-chain C2 and C3-organosulfonates (16–20), which are excreted and subsequently utilized by secondary consumer organisms (17, 21–23). These secondary organisms—facultative anaerobes or aerobes—cleave the C–S bond using sulfolyases and release sulfite to enable carbon assimilation (24, 25). In anaerobic environments, short-chain organosulfonates serve as terminal electron acceptors or sulfur sources, a process facilitated by glycyl radical enzymes (26). In contrast, sulfolytic bacteria can directly cleave the C–S bond in SQ, releasing sulfite and converting the entire carbon backbone to glucose for entry into central carbon metabolism (19, 27).

Although SQ metabolism is well-characterized, much less is known about other sulfosugars. One such compound is sulfofucose (SFuc, 6-deoxy-6-sulfo-D-galactose), a rare sulfosugar reported in the glycolipids of algae from the order *Fucaceae*, which are distributed across the North Atlantic Ocean (28), and in cell surface N-linked glycans from the extremophile archaeon *Thermoplasma acidophilum* (Fig. 1b) (29). SFuc differs from sulfoquinovose only in the configuration of carbon-4. Despite this minimal structural difference, no biosynthetic or catabolic pathways for SFuc have been identified.

Here, we report the isolation of *Paracoccus wurundjeri sp. nov*. strain Merri, a bacterium capable of using SFuc as its sole carbon source. Using uniformly ^13^C-labeled SFuc, we show that the entire carbon skeleton is metabolized and that sulfite is released as the end product. Comparative proteomics reveals increased abundance of proteins associated with organosulfur metabolism during growth on SFuc. Metabolomics analysis of cell lysates identified a pathway intermediate consistent with an Entner–Doudoroff (ED)-like pathway for SFuc metabolism to sulfolactaldehyde (SLA), and a sulfolyase pathway for biomineralization via SL. Finally, recombinant expression and *in vitro* reconstitution of four enzymes allowed demonstration of the individual steps of a sulfofucolytic ED pathway that converts SFuc to SLA and pyruvate. These findings support the operation of a tandem pathway comprising oxidative cleavage of SFuc to SLA, followed by canonical biomineralization to sulfite via SL sulfolyase.

## RESULTS

### Isolation and characterization of a *Paracoccus wurundjeri* bacterium able to grow on sulfofucose

**Fig 2:**
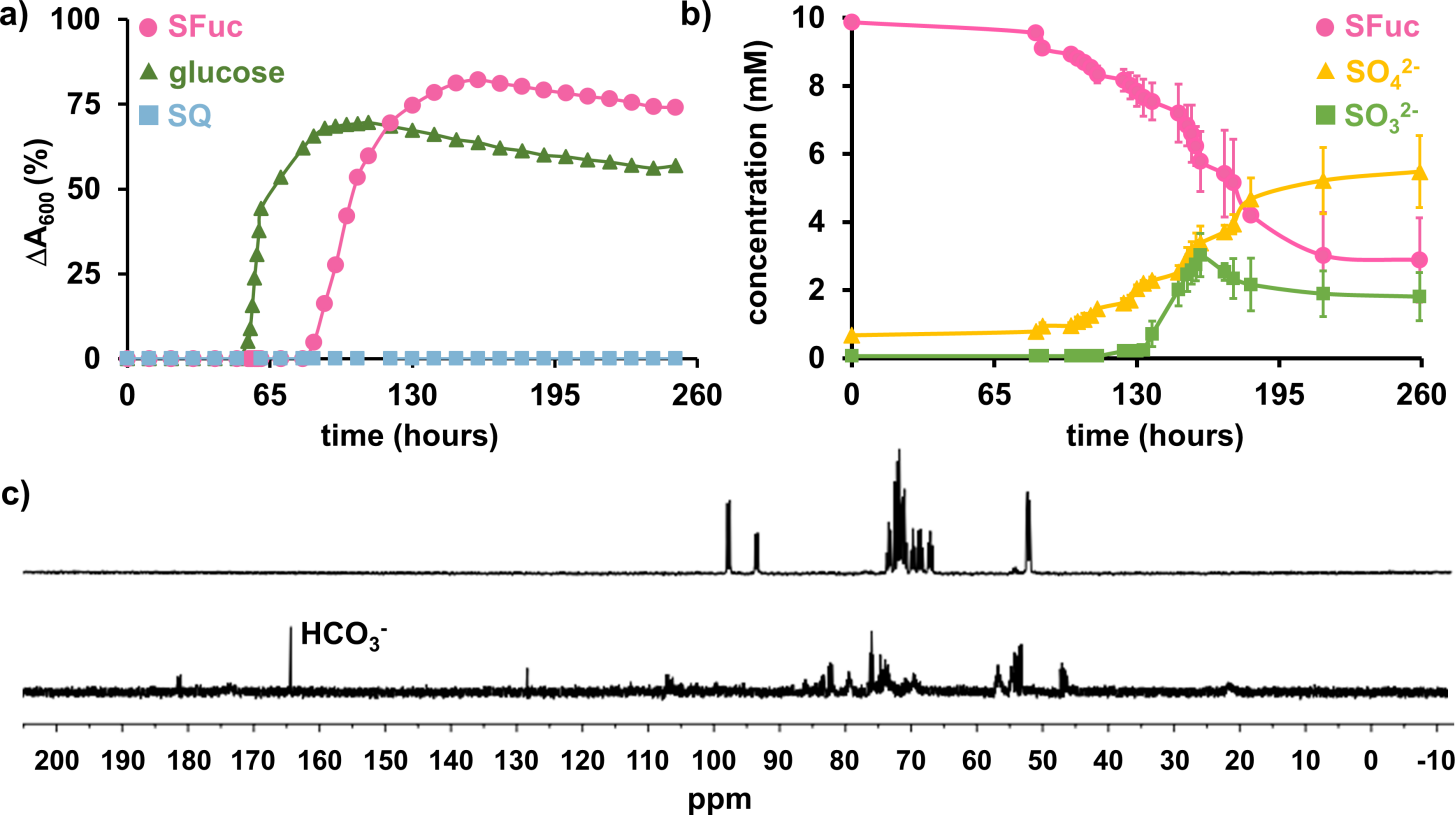
*P. wurundjeri* strain Merri grows on SFuc as a sole carbon source. (**a**) Growth of strain Merri on glucose, SFuc, and SQ. Turbidity was measured at A_600_ nm, and the cells were cultured in M9 minimal media containing 5 mM of each carbon source, supplemented with a vitamin mix and trace metals. (**b**) Strain Merri uses SFuc as a carbon source and produces sulfite. Independent replicates show the change in concentration of SFuc, sulfite, and sulfate throughout the growth of strain Merri on SFuc. Analyses were performed in duplicate, and errors are reported as standard deviations. (**c**) ^13^C NMR spectra of M9 minimal media containing 10 mM ^13^C_6_-SFuc (top) and after 5 days of growth of strain Merri at the mid-log phase (bottom).

A soil sample from Joe’s Garden, an inner-Melbourne creekside market garden that has been continuously farmed by Chinese and Italian gardeners for 150 years, was used to inoculate a vitamin-enriched defined medium containing 10 mM SFuc as the sole carbon source. Sequential subculturing, along with repeated plating onto SFuc-containing media agar plates, followed by regrowth in SFuc liquid media, led to the isolation of strain Merri. This strain grew on glucose and SFuc to similar peak absorbance but did not grow on SQ (Fig. 2a). Genomic DNA was extracted from strain Merri and was sequenced using the Illumina NextSeq platform. Analysis of the 16S rRNA gene against the NCBI 16S rRNA database (30) revealed the most similar sequence to be from *Paracoccus methylarcula* strain H1 (97.18% identity). With 97% 16S rRNA sequence identity generally recognized as the cutoff for an organism to be considered the same species, this places strain Merri firmly within the genus *Paracoccus* but close to being considered a new species. In light of this, we performed *in silico* analysis using DNA–DNA hybridization against the Genome Taxonomy Database. This analysis showed that strain Merri had 94.37% average nucleotide identity (ANI) to the most closely related reference genome (*Paracoccus* sp. 003591515), which is below the threshold to be considered the same species (95% ANI) (31, 32). Given that ANI is generally considered a more robust method to define taxonomic lineages (31, 33), we propose that strain Merri represents a new member of the genus *Paracoccus*, which we have designated *P. wurundjeri sp. nov*. (Strain ID: Merri), based on its isolation from the banks of Merri Creek, which runs through the lands of the indigenous Wurundjeri people. General features of the draft genome are shown in Table 1.

**TABLE 1:** Key features of *P. wurundjeri* strain Merri (GenBank accession: Bioproject PRJNA993841) draft genome assembly

	Strain Merri
Genome size	5,082,950
Number of contigs	74
GC %	60.1
Number of ORFs	4,913
Number of putative genes	4,966
Number of putative tRNA	49
Number of putative rRNA	3
Number of putative tmRNA	1
Number of genes assigned a function (%)	2,847 (57%)

To trace SFuc catabolism, we examined the fate of its carbon and sulfur components upon metabolism by *P. wurundjeri* strain Merri. To examine the fate of sulfur, the spent culture media from strain Merri grown on SFuc were analyzed. SFuc consumption was tracked using a reducing sugar assay, whereas sulfite and sulfate productions were quantified with colorimetric and turbidometric assays, respectively. Most SFuc was consumed over 200 h, accompanied by the production of sulfate and sulfite (Fig. 2b). Sulfite levels peaked at 160 h before declining, a pattern consistent with the known oxygen sensitivity of sulfite (27). This suggests that sulfite is initially produced and subsequently undergoes autooxidation to sulfate. ^13^C NMR analysis of the culture medium of strain Merri grown to mid log-phase (5 d) on ^13^C_6_-SFuc revealed complete consumption of the substrate and production of ^13^C-bicarbonate (Fig. 2c); extended growth to stationary phase (14 d) revealed no distinct carbon-containing compounds, indicating complete utilization of the complete carbon chain (data not shown).

### Identification of genetic loci expressed upon growth on sulfofucose

**Fig 3:**
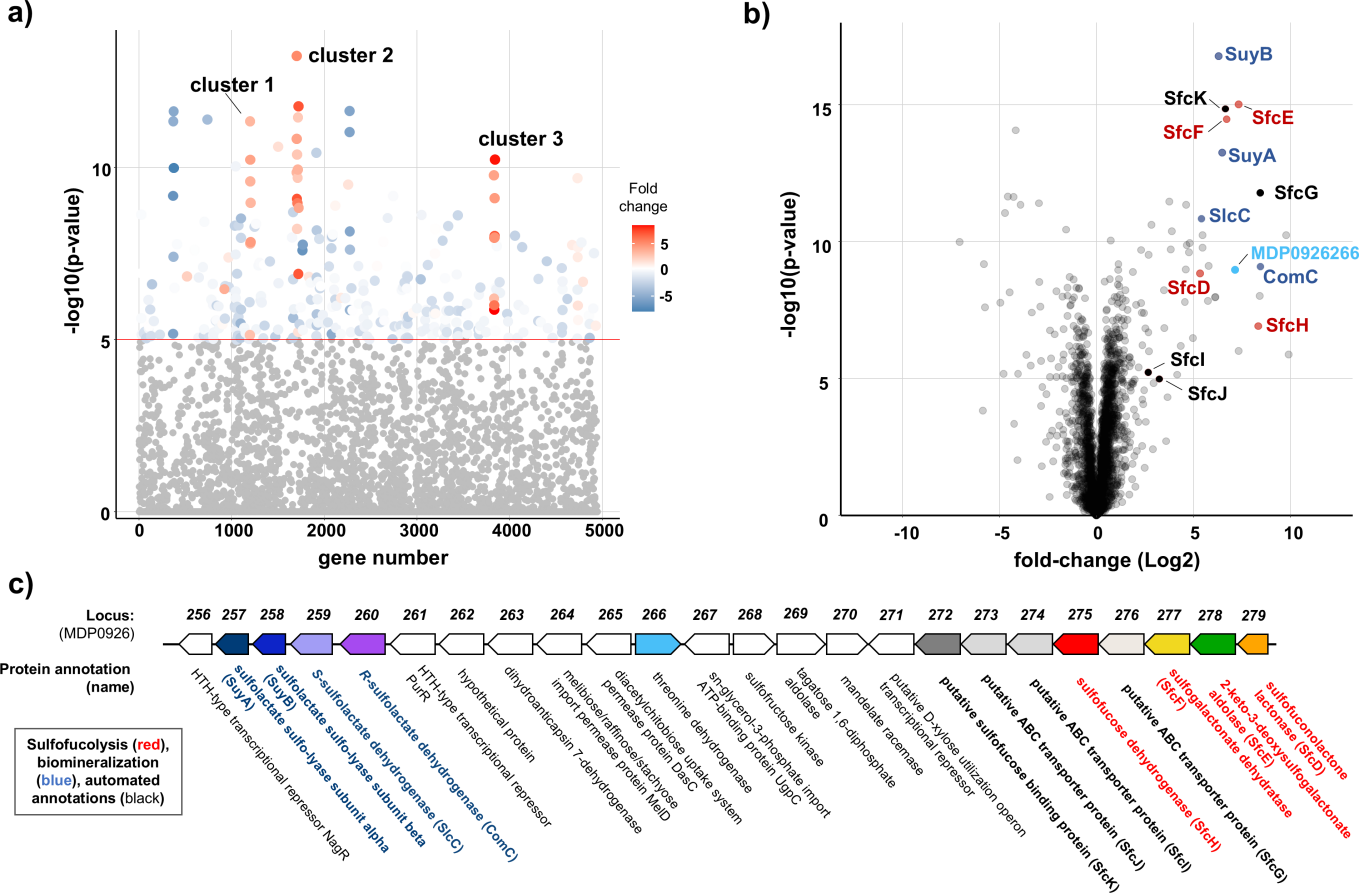
Genome and proteome analyses reveal a gene cluster for organosulfonate metabolism in *P. wurundjeri* strain Merri. The cells were cultured in vitamin-supplemented minimal media with either SFuc or glucose and analyzed via comparative proteomics. (**a**) Manhattan plot highlighting three differentially expressed gene clusters. (**b**) Volcano plot showing proteins identified in the SFuc degradation pathway (red, SfcD, SfcE, SfcF, SfcG; blue, SlcC, ComC, SuyAB; and black, putative SFuc binding protein and ABC transporter cassette). (**c**) Gene neighborhood diagram of "cluster 2," associated with organosulfonate metabolism. Genes annotated in red are sulfofucolytic genes; in blue are putative biomineralization genes; in bold black text are a putative SFuc binding protein and ABC transporter cassette; and in plain black text are automated genome annotations.

To identify proteins potentially involved in SFuc metabolism, we cultured *P. wurundjeri* strain Merri using either SFuc or glucose as the sole carbon source and analyzed the resulting changes in the proteome (Fig. 3a and b). Growth on SFuc led to significant increases in the abundance of proteins primarily encoded within three genomic regions, designated clusters 1–3.

Cluster 1 (Locus IDs MDP0925965–0925971) comprises seven genes, all of which showed increased protein abundance (Fig. S1). These genes encode enzymes annotated for roles in gluconeogenesis and the pentose phosphate pathway. Sulfoglycolysis circumvents the glycolytic steps that generate glucose-6-phosphate and fructose-6-phosphate—key intermediates required for the pentose phosphate pathway and the biosynthesis of sugar nucleotides and cell wall components. Previous studies in *E. coli* have shown that sulfoglycolysis via the sulfo-EMP pathway elevates gluconeogenic flux due to the generation of the C3-phosphate DHAP (34); similarly, a sulfofucolytic pathway in *P. wurundjeri* would require gluconeogenesis to supply hexose-phosphate intermediates from DHAP or pyruvate. Cluster 3 (MDP0926751–0926761) includes nine proteins with significantly increased abundance, associated with fumarate and tartrate metabolism, which may be linked to the Krebs cycle (Fig. S1). This cluster also harbors gene MDP0926757, annotated as a hypothetical protein but homologous to the TauE transmembrane sulfite exporter (35).

Cluster 2 (MDP0926256–0926279) contains 24 genes, many of which were increased in abundance in SFuc-grown cells (Fig. 3c, Table S1). Some of these genes are annotated with functions related to organosulfonate metabolism and belong to protein families (PFAM) associated with SL biomineralization in *Chromohalobacter salexigens* (25), *Paracoccus pantotrophus* (36), and *Desulfvibrio* sp. DF1 (37) (Table S2). Among the proteins of increased abundance were homologs of SL dehydrogenases SlcC (MDP0926259) and ComC (MDP0926260), which could act as a two-component SL racemase (Fig. S5 and 6) (25). Additionally, MDP0926258, annotated as SL sulfolyase B (SuyB), and MDP0926257, encoding a short protein homologous to SL sulfolyase A (SuyA) (Fig. S7 and 8) (36, 37), were increased in abundance. Finally, protein MDP0926266, annotated as threonine dehydrogenase, was present in increased abundance and is a candidate for an SLA dehydrogenase for the oxidation of SLA to SL. Together, these proteins may form a C3-organosulfonate biomineralization pathway that involves the oxidation of *S*-sulfolactaldehyde to *S*-sulfolactate, catalyzed by MDP0926266, racemization to *R*-sulfolactate catalyzed by SlcC/ComC, and SuyAB-catalyzed cleavage of *R*-sulfolactate to yield pyruvate and sulfite, with the latter exported via TauE.

Turning to the initial steps of SFuc metabolism, several genes within cluster 2 are homologous to those involved in known sulfosugar breakdown pathways. Two groups of interest were identified. The first group includes homologs of the sulfo-EMP pathway proteins: MDP0926268 and MDP0926269, belonging to PF00294 and PF01791, respectively, protein families encompassing sulfofructose kinase and sulfofructose-1-phosphate aldolase (Table S3) (17). Possibly, these could act on the 4-epimeric SFuc-derived intermediates, such as the sulfoketohexose sulfotagatose (6-deoxy-6-sulfo-D-tagatose) and sulfotagatose-1-phosphate, with the latter enzyme MDP0926269 potentially producing SLA and DHAP. Although no aldose–ketose isomerase is encoded within this cluster, a homolog is encoded elsewhere in the genome (protein MDP0925592) belonging to protein family PF07221, the same family as SQ-sulfofructose isomerase within the sulfo-EMP pathway (17). However, none of these three proteins showed significantly increased abundance in SFuc-grown cells.

The second group of genes of interest from cluster 2 includes MDP0926275, MDP0926278, and MDP0926279—the corresponding proteins of which were all increased in SFuc-grown cells and are associated with PF13561, PF03328, and PF09450, respectively. These protein families include enzymes from the sulfoglycolytic ED pathway: SQ dehydrogenase, 2-keto-3-deoxysulfogluconate (KDSG) aldolase, and sulfogluconolactone (SGL) lactonase, respectively (Table S4) (18). A fourth enzyme required for a complete sulfoglycolytic ED pathway, SG dehydratase (belonging to PF00920), was not encoded within cluster 2 or elsewhere in the genome. However, the protein encoded by MDP0926279 in cluster 2 was increased in abundance and belongs to protein families PF13378 and PF02746, families to which D-galactonate dehydratase belongs. Notably, D-galactonate bears a close structural resemblance to sulfogalactonate (SGal). Thus, we propose that protein MDP0926279 may be capable of dehydrating a sulfofucolytic ED intermediate such as SGal. Associated with these genes was a likely ABC transporter cassette and substrate binding protein, which we assign as a putative SFuc importation system.

Collectively, these data support the existence of either an EMP-like or ED-like sulfofucolysis pathway. In the proposed sulfofucolytic EMP pathway, SFuc would be isomerized to sulfotagatose (by protein MDP0925592), phosphorylated to sulfotagatose-1-phosphate (by protein MDP0926268), and then cleaved via a retroaldol reaction (by protein MDP0926269) to yield SLA and DHAP. Alternatively, a sulfofucolytic ED pathway could involve the oxidation of SFuc to sulfogalactonolactone (SGalL) (by protein MDP0926275), followed by lactone hydrolysis to form SGal (by protein MDP0926279). This intermediate would undergo dehydration to 3-deoxy-2-ketosulfogalactonate (KDSGal) (by protein MDP0926277), and retroaldol cleavage (by protein MDP0926278) to generate *S*-sulfolactaldehyde and pyruvate. In either pathway, *S*-sulfolactaldehyde would be further metabolized by oxidation to *S*-sulfolactate, and then the SlcC/ComC–SuyAB biomineralization system encoded in cluster 2 would convert it to sulfite, which would be exported by TauE homolog protein MDP0926575 in cluster 3. Meanwhile, DHAP or pyruvate would enter central carbon metabolism.

### Cell-free lysate analysis reveals sulfofucose-stimulated dehydrogenase activity

To explore enzymatic activities in SFuc catabolism, cell-free lysates were prepared from *P. wurundjeri* strain Merri grown in minimal medium with 5 mM SFuc. Cells were harvested in mid-log phase, lysed by sonication, and clarified by centrifugation. NAD(P)^+^-dependent dehydrogenase activity was assessed by monitoring NAD(P)H formation at 340 nm. An increase in absorbance was observed only when lysate and SFuc were present, indicating SFuc-stimulated dehydrogenase activity (Fig. S2). To test for isomerase activity, ^1^H NMR spectroscopy was used to monitor reactions in lysate supplemented with recombinant shrimp alkaline phosphatase, which prevented phosphorylation and potential downstream turnover of sulfotagatose. After 24 h of incubation with SFuc, no new resonances consistent with sulfotagatose were detected (data not shown), suggesting the absence of a SFuc–sulfotagatose isomerase. These findings support an ED-like sulfofucolytic pathway in which SFuc undergoes initial NAD(P)^+^-dependent oxidation.

### Detection of sulfogalactonate and sulfolactate by metabolomics analyses

To investigate the metabolic fate of SFuc in *P. wurundjeri* strain Merri, we performed metabolomic analysis on cell lysates from cultures grown with SFuc as the sole carbon source. Using ion chromatography-mass spectrometry (IC-MS), we screened for intermediates indicative of sulfofucolytic EMP- or ED-like pathways. Metabolites were extracted from mid-log phase cell lysates and were analyzed using ion chromatography coupled to high-resolution mass spectrometry.

**Fig 4:**
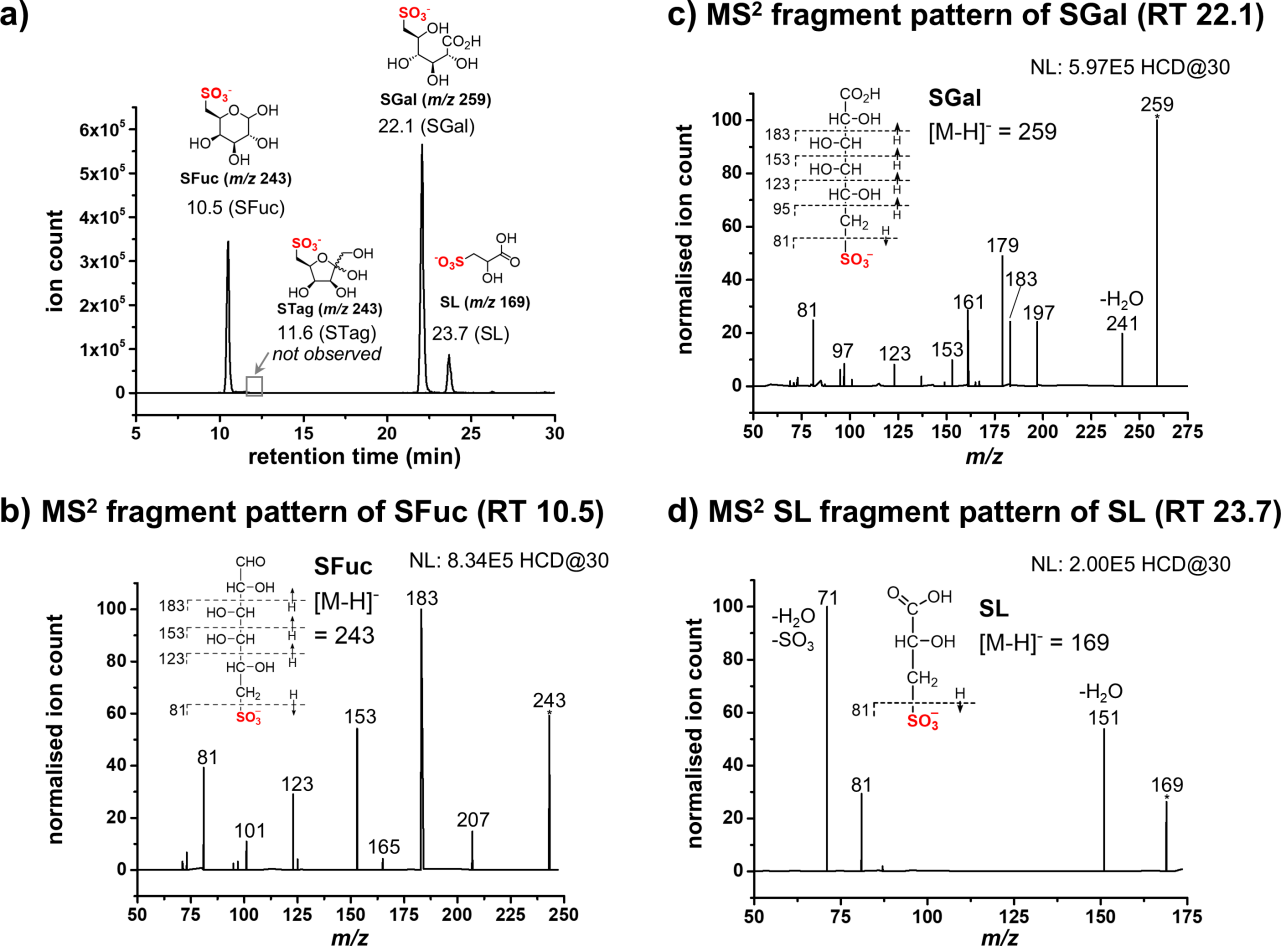
Metabolomic analysis of *P. wurundjeri* strain Merri grown on sulfofucose. Strain Merri cells were grown on SFuc until mid-log phase, harvested, lysed, and metabolites were extracted from cells. Targeted LC-IC-MS was employed, and potential sulfofucolytic metabolites were screened for using SIM scans. (**a**) Extracted ion chromatograph showing key organosulfur metabolites within *P. wurundjeri* cells. (**b**) MS^2^ fragment pattern of SFuc. (**c**) MS^2^ fragment pattern of SGal. (**d**) MS^2^ fragment pattern of SL.

In SFuc-grown samples, we observed peaks with retention times, *m/z* values, and fragmentation patterns matching those of authentic standards for sulfogalactonate (SGal, *m/z* 259) and SL (*m/z* 169) (Fig. 4; Fig. S3). In contrast, no signal corresponding to sulfotagatose, an expected intermediate of an EMP-like pathway, was detected, consistent with the lack of SFuc–sulfotagatose isomerase activity in lysate assays. The detection of SGal supports an ED-like pathway, where SFuc is oxidized to sulfofuconolactone, followed by hydrolysis to yield SGal. The presence of SL, the product of SLA oxidation, supports downstream metabolism involving an SLA dehydrogenase and the SlcC/ComC–SuyAB biomineralization pathway to produce sulfite, detected in the colorimetric assay. Combined with SFuc-stimulated NAD(*P*)^+^-dependent dehydrogenase activity, this supports the operation of an ED-like sulfofucolytic pathway in *P. wurundjeri*.

### *In vitro* demonstration of an Enter-Doudoroff pathway for sulfofucose

**Fig 5:**
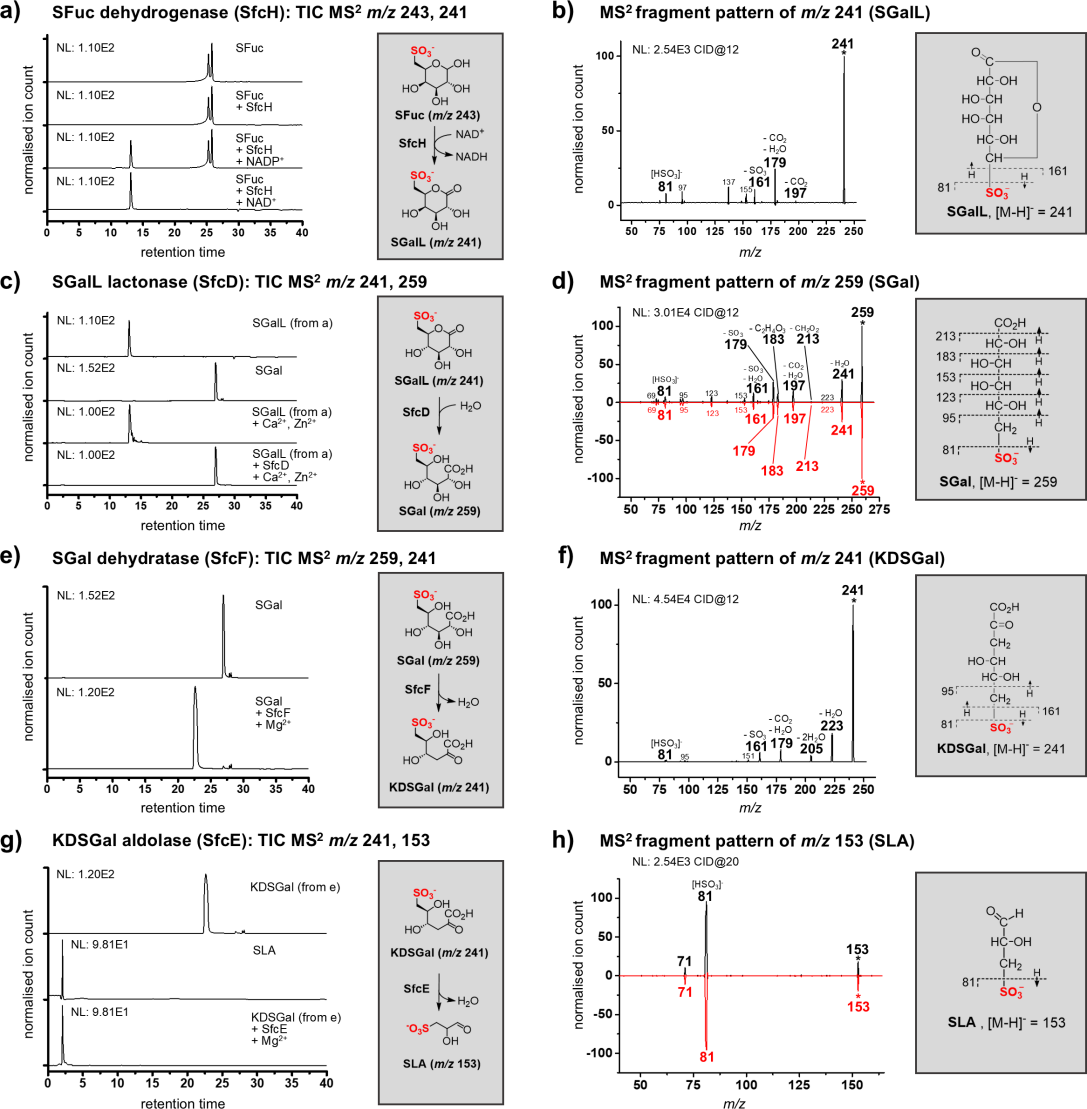
*In vitro* reconstitution of individual steps of the sulfofucolytic ED pathway. The enzymatic conversions of SFuc to SGalL, SGalL to SGal, SGal to KDSGal, and KDSGal to SLA, catalyzed by SfcH, SfcD, SfcF, and SfcE, respectively. Targeted MRM-MS analysis was used to monitor analytes. (**a**) Ion chromatograms of SFuc (*m/z* 243) and assays containing SFuc with SfcH and NAD(P)^+^. (**b**) MS^2^ fragment pattern of SGalL *m/z* 241. (**c**) Ion chromatograms of assays containing SGalL treated with SfcD, CaCl_2_, and ZnCl_2_. (**d**) MS^2^ fragment pattern of experimentally obtained SGal (black) and chemically synthesized (red) *m/z* 259. (**e**) Ion chromatograms of SGal and assays containing SGal with SfcF and MgCl_2_. (**f**) MS^2^ fragment pattern of KDSGal *m/z* 241. (**g**) Ion chromatograms of assays containing KDSGal treated with SfcE and MgCl_2_. (**h**) MS^2^ fragment pattern of experimentally obtained SLA (black) and chemically synthesized (red) *m/z* 153. Pyruvate, the second product of the SfcE reaction, was confirmed by ion chromatography-mass spectrometry (Fig. S4).

To demonstrate the biochemical steps of sulfofucolysis, genes encoding the candidate enzymes were heterologously expressed in *E. coli*, and the corresponding proteins were purified. Based on the roles subsequently demonstrated in SFuc catabolism, these genes were renamed *sfcH* (MDP0926275; dehydrogenase), *sfcD* (MDP0926279; lactonase), *sfcF* (MDP0926277; dehydratase), and *sfcE* (MDP0926278; aldolase), reflecting their membership in a sulfofucose degradation pathway. Incubation of SfcH with SFuc and either NADP^+^ or NAD^+^ produced a compound with *m/z* 241, two mass units lower than SFuc, consistent with oxidation to SGalL (Fig. 5). MS^2^ fragmentation of this species matched that of the diastereoisomer SGL (18). Notably, this enzyme displayed greater activity on NAD^+^, a preference that matches that of SQ dehydrogenase from *P. putida* (38). Subsequent addition of SfcD along with Ca^2+^ and Zn^2+^ converted the oxidized intermediate into a compound with identical retention time and MS^2^ fragmentation pattern as authentic chemically synthesized SGal. When chemically synthesized SGal was incubated with SfcF, a new species with *m/z* 241 (18 mass units lower than sulfogalactonate) was detected, consistent with conversion to KDSGal, and which displayed a fragmentation pattern consistent with the diastereoisomer KDSG (18). Finally, the addition of SfcE and Mg^2+^ to this reaction mixture yielded a product with retention time and MS^2^ fragmentation matching that of chemically synthesized SLA. Separately, analysis of this reaction mixture by IC-MS demonstrated formation of pyruvate (Fig. S4). SfcE was inactive when supplemented with Mg^2+^ or Mn^2+^.

### Identification of possible sulfofucolytic pathways in proteobacteria

**Fig 6:**
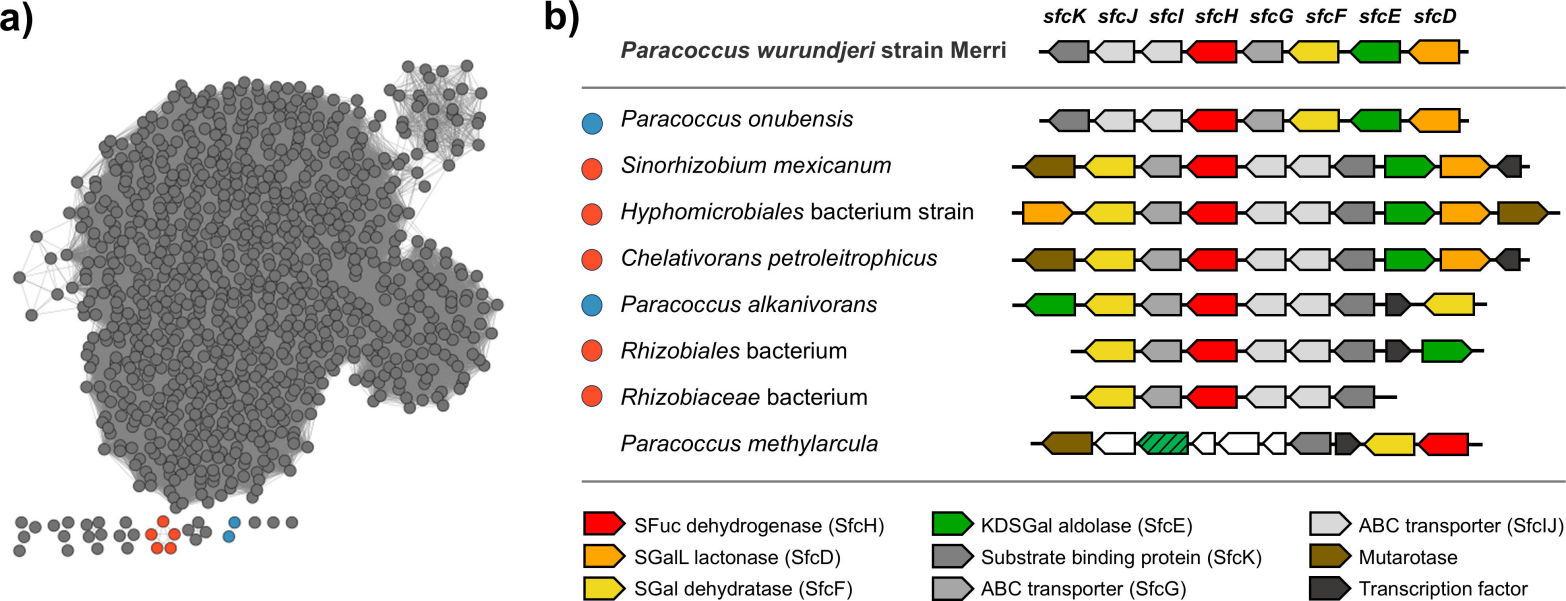
Proposed sulfofucolytic ED pathway gene clusters distributed across assorted proteobacteria. (**a**) Sequence similarity network (SSN) of SGal dehydratase SfcF homologs at an alignment score of 160. Colored clusters represent organisms harboring gene clusters containing homologues of sulfofucolytic ED pathway genes *sfcE*, *sfcF,* and *sfcH*. (**b**) Gene clusters encoding sulfofucolytic ED pathways. Colored circles at left correspond to colored clusters in part (**a**). UniProt protein accession codes for SfcF homologs are as follows: *P. wurundjeri* strain Merri (MDP0926277), *Paracoccus onubensis* strain L29414 (A0A418T7C2), *Sinorhizobium mexicanum* strain ITTG R7 (A0A859QZ22), *Hyphomicrobiales* bacterium strain SCN_18_10_11_15_R4_B_69_13 (A0A8I1P1S0), *Chelativorans petroleitrophicus* strain SCAU2102 (A0A9 × 2XAH4), *Paracoccus alkanivorans* strain 4-2 (A0A3M0MGN3), *Rhizobiales* bacterium strain 65-79 (A0A1M2Z1S0), *Rhizobiaceae* bacterium strain FW021_bin.52 (A0A522R0Q2), and *Paracoccus methylarcula* strain H1 (A0A3R7NEB5).

To identify candidate sulfofucolytic ED pathways in other bacteria, we used tools from the Enzyme Function Initiative (EFI) (39, 40) and performed a BLAST search using the sequence of sulfogalactonate (SGal) dehydratase SfcF as a query. Homologous sequences were retrieved and used to construct a sequence similarity network (Fig. 6). We then examined the genome neighborhoods of these SfcF homologs for the presence of genes encoding additional enzymes associated with the sulfofucolytic ED pathway, specifically, those from PFAM families PF08450 (SGalL lactonase; SfcD), PF13378-PF02746 (SGal dehydratase, SfcF), and PF03328 (KDSGal aldolase, SfcE) (Table S5). This analysis identified two clusters of proteins. Gene neighborhood diagrams from the parent bacteria showed that some clusters encode all four core enzymes of the proposed pathway, along with an ABC transporter system. Other clusters lacked an obvious lactonase candidate, and some encoded an aldose-1-epimerase, which may function as a SFuc mutarotase. Many of these bacteria also possessed genes encoding homologs of ComC, SlcC, SuyA, and SuyB, suggesting that they also possess the capacity for complete metabolism of SFuc through a tandem sulfofucolytic-biomineralization pathway (Table S6). Notably, the closest relative of *P. wurundjeri* strain Merri, based on 16S rRNA analysis, *P. methylarcula* strain H1, contains syntenic sulfofucolytic and biomineralization gene clusters.

### A missing step in SLA oxidation to SL

Proteomic and genomic analyses did not identify a homolog of GabD/SlaB capable of oxidizing SLA, the product of KDSGal aldolase (SfcE) activity on KDSGal. However, metabolomic analysis revealed the accumulation of SL as an intracellular metabolite during the growth of *P. wurundjeri* on SFuc. Within gene cluster 2, comparative proteomics highlighted putative threonine dehydrogenase MDP0926266, which we considered as a candidate SLA dehydrogenase. However, the recombinantly expressed MDP0926266 protein showed no activity toward SLA in the presence of either NAD^+^ or NADP^+^.

## DISCUSSION

**Fig 7:**
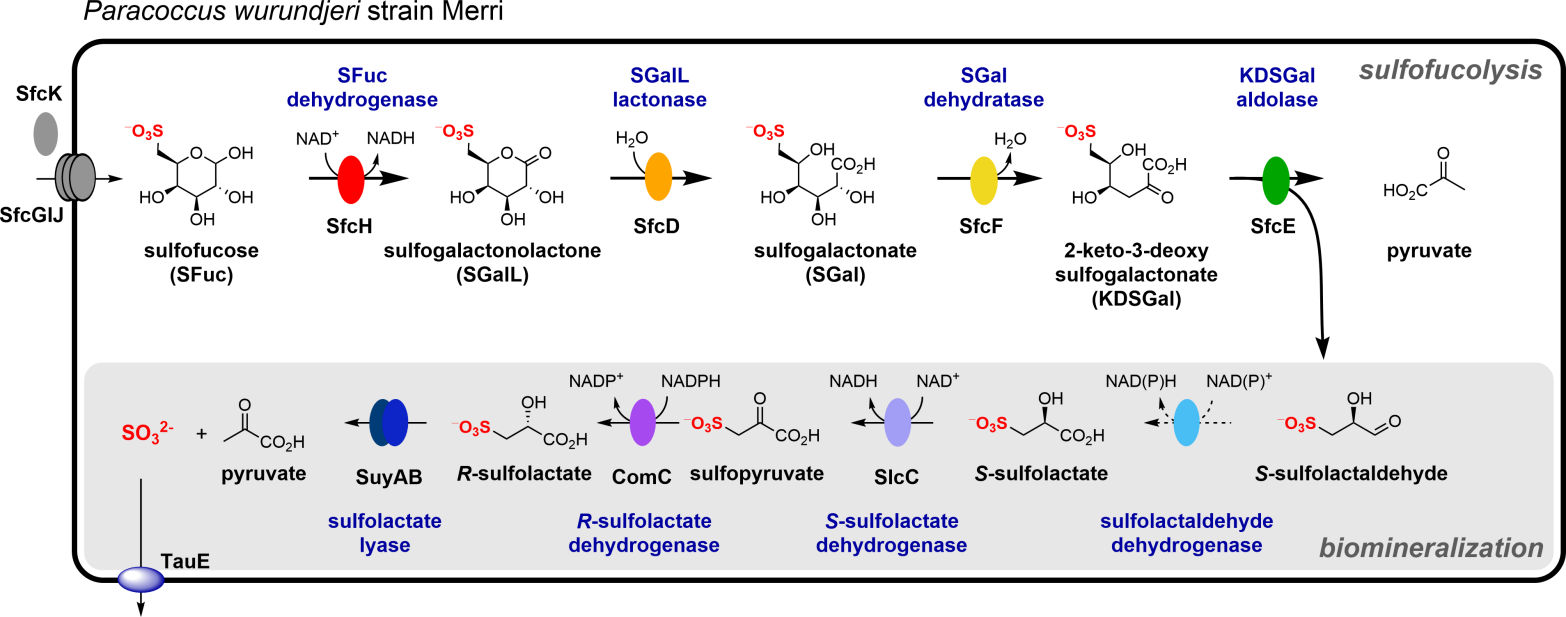
Sulfofucolytic and proposed biomineralization pathway within *P. wurundjeri* strain Merri. The experimentally demonstrated sulfofucolytic steps (bold arrows) involve SFuc dehydrogenase (SfcH), which catalyzes the conversion of SFuc to SGalL; SGalL lactonase (SfcD), which catalyzes the hydration of SGalL to SGal; SGal dehydratase (SfcF), which catalyzes the dehydration of SGal to KDSGal; and KDSGal aldolase (SfcE), which catalyzes the lysis of KDSGal to yield SLA and pyruvate. The resultant SLA is proposed to undergo a biomineralization process in which *S*-SLA is oxidized to *S*-SL by an unknown SLA dehydrogenase (dotted arrow), racemized by SL dehydrogenases (SlcC/ComC) to *R*-SL, and finally, sulfite is liberated by SL lyase (SuyAB) (plain arrows).

In this study, we identify a novel bacterium, *P. wurundjeri sp. nov*., which can grow on the rare sulfosugar SFuc as the sole carbon source and effects its complete degradation to sulfite, via SGal and SL. This organism utilizes a sulfofucolytic Entner–Doudoroff (ED) pathway that cleaves the C6 backbone of SFuc into two C3 fragments: SLA and pyruvate (Fig. 7). SLA is then biomineralized through a pathway involving oxidation to SL, and then sulfolyase catalyzes the elimination of sulfite to give pyruvate. The pyruvate outputs of this pathway are used in central carbon metabolism, whereas the sulfite is excreted into the culture media, where it can undergo autooxidation to sulfate. This finding adds to the known metabolic versatility of the genus *Paracoccus*, as *P. pantotrophus* strain NKNCYSA has previously been reported to degrade a range of organosulfonates, including cysteate, taurine, homotaurine, isethionate, and SL (41), suggesting a broad organosulfonate catabolic potential within this taxonomic grouping. However, BLAST analysis reveals that *P. pantotrophus* NKNCYSA does not contain a candidate sulfofucolytic pathway.

The observation of SGal by metabolomic analysis of SFuc-grown *P. wurundjeri* cells is diagnostic of an oxidative sulfofucolytic pathway. We identified four enzymes (SfcH, SfcD, SfcF, and SfcE) that collectively enable the stepwise conversion of SFuc through an Entner–Doudoroff–like pathway: SFuc oxidation to SGalL (SfcH), hydrolysis to SGal (SfcD), dehydration to KDSGal (SfcF), and aldol cleavage to yield SLA and pyruvate (SfcE). These steps mirror the enzymatic steps of the canonical sulfoglycolytic ED pathway used for SQ catabolism, with the key distinction to SFuc being the stereochemistry at C4 of the sugar. This renders their corresponding intermediates epimeric: SGalL vs. SGL, SGal vs. SG, and KDSGal vs. KDSG. Despite this difference, the aldolase-catalyzed cleavage of both KDSG and KDSGal generates the same *S*-enantiomer of SLA. This is because the stereocenter in SLA originates from carbon 5, which is common to both sulfosugars, whereas the configuration at carbon 4 is lost during cleavage, and so does not affect the stereochemistry of the product.

All but one of the enzymes in the sulfofucolytic ED pathway belong to the same protein families as those in the canonical sulfoglycolytic ED pathway for sulfoquinovose catabolism (18). The key difference lies in the dehydratase step: while sulfoquinovose degradation employs SG dehydratase (PF00920), the sulfofucolytic pathway utilizes SGal dehydratase, which belongs to a distinct pair of families (PF13378, PF02746). This divergence provided a basis for a targeted bioinformatic search, enabling the identification of related gene clusters in other bacteria. Using this signature, we identified a small subset of Proteobacteria harboring syntenic gene clusters that encode homologs of the sulfofucolytic ED pathway. Notably, like that of strain Merri, all of these gene clusters were associated with ABC transporter systems, which may mediate SFuc uptake. ABC transporters have previously been implicated in SQ uptake in sulfo-ED pathways (18, 42), and SQ-binding proteins associated with ABC transporters have been characterized biochemically and structurally (27, 43, 44). Several organisms contained a candidate SFuc mutarotase, which could facilitate the oxidation of SFuc to SGalL if the SFuc dehydrogenase displays specificity for one of the anomers of SFuc by accelerating the rate of anomer interconversion. Notably, anomeric preference has been reported in the first step of the sulfo-EMP pathway in *E. coli*, where SQ isomerase preferentially acts on the β-anomer of SQ during its isomerization to sulfofructose (45).

Interestingly, several of the candidate sulfofucolytic organisms lacked an obvious lactonase homolog, raising the possibility that they utilize a variant of the pathway that bypasses the lactone hydrolysis step. This difference is reminiscent of the De Ley–Doudoroff pathway—a variant of the Entner–Doudoroff pathway—in which D-galactose is oxidized by a NAD^+^-dependent dehydrogenase to D-galactonate, followed by dehydration to 2-keto-3-deoxygalactonate and subsequent retro-aldol cleavage to yield pyruvate and glyceraldehyde (46, 47). By analogy, these bacteria may encode a lactonase-independent sulfofucolytic ED pathway in which SFuc is directly oxidized to SGal, thereby bypassing the need for SGalL hydrolysis.

Comparative proteomics of strain Merri identified a cluster of genes that encoded homologues of proteins capable of the biomineralization of the C3-organosulfonate SL. Specifically, these genes encode homologues of the two-component NAD(P)^+^-dependent SL racemases SlcC/ComC (25), and SL lyase (SuyAB), which cleaves SL to yield pyruvate and sulfite (36, 37). Although no homologs of classical SLA dehydrogenases SlaB/GabD (18, 19, 48) were present, a candidate SLA dehydrogenase (MDP0926266) was identified that belonged to the protein families PF08240-PF00107 and was annotated as threonine dehydrogenase; however, recombinant MDP0926266 did not oxidize SLA. Nonetheless, metabolomic analysis of lysates from SFuc-grown cells revealed the presence of SL, consistent with the existence of this step in the pathway. Together, these data provide support for a pathway involving SLA dehydrogenase and the SlcC/ComC–SuyAB biomineralization pathway, proceeding via the sequence *S*-SLA → *S*-SL → sulfopyruvate → *R*-SL → pyruvate + sulfite. Genes encoding homologs of SlcC, ComC, SuyA, and SuyB were present in several of the bacterial strains encoding candidate sulfofucolytic pathways, namely, *Paracoccus alkanivorans*, *Rhizobiaceae* sp., *Sinorhizobium mexicanum*, *Chelativorans petroleitrophicus*, and *Paracoccus onubensis*, suggesting that these bacteria also utilize tandem sulfofucolytic-biomineralization pathways (Table S6).

This work provides evidence for the coexistence of both sulfofucolysis and biomineralization pathways within a single bacterium. Coexistence of a sulfofucolytic/sulfoglycolytic and biomineralization pathway in a single bacterium is unusual. One example involves an environmental *Klebsiella* sp., which was shown to degrade SQ to SL and, after 24 h growth, produced trace amounts of sulfate—equivalent to approximately 10% of the initial SQ (49). However, the molecular mechanisms underlying these observations in the *Klebsiella* strain remain unresolved.

The occurrence of SFuc has only been documented in two reports: within glycoconjugates produced by *Fucaceae* algae and an extremophile archaeon (28, 29). This limited evidence suggests that SFuc may be relatively rare in the environment. However, members of the *Fucaceae* are widespread in coastal regions around the North Atlantic Ocean, raising the possibility that SFuc is more common than currently recognized. Moreover, SFuc shares an identical molecular formula with sulfoquinovose (SQ), meaning it could have been misidentified in studies that relied exclusively upon mass spectrometry for identification. If so, its true environmental distribution may be broader than literature reports suggest.

Strain Merri was isolated from Joe’s Garden, a riparian market garden located on the banks of Merri Creek in Melbourne, Australia. Given the site’s history of flooding, the bacterium, or its substrate, may have a soil or aquatic origin. The Proteobacteria harboring syntenic sulfofucolytic gene clusters have been isolated from soil and oil-rich environments and include rhizobia, indicating that SFuc may be encountered in soil ecosystems, particularly within the rhizosphere. In these niches, bacteria may encounter SFuc released from known or yet-unidentified SFuc-containing glycoconjugates. Although no candidate sulfofucosidases were identified in this study, previous work has shown that sulfoquinovosidases from *E. coli* and *Agrobacterium tumefaciens* are inactive on PNP α-sulfofucoside (15), suggesting limited enzymatic cross-reactivity and the potential for discovery of a novel sulfofucosidase. Finally, we highlight the existence of another sulfohexose, sulforhamnose, which arises transiently as an intermediate in the sulfo-EMP pathway from SQ and is subsequently isomerized back to SQ or sulfofructose, key intermediates in that pathway (45).

In conclusion, this work identifies a bacterium, *P. wurundjeri*, capable of complete catabolism of the rare sulfosugar SFuc. We provide evidence for a two-phase metabolic route: an oxidative sulfofucolytic Entner–Doudoroff (ED) pathway that cleaves SFuc into C3-organosulfonate intermediates, and a downstream biomineralization pathway involving SL lyase–mediated conversion of SL to sulfite. Although most enzymes in the sulfofucolytic ED pathway share homology with those in the known sulfoglycolytic ED pathway, the dehydratase step is catalyzed by a protein from a distinct family (PF13378, PF02746). This divergence enabled the identification of a small set of bacteria predicted to possess similar SFuc catabolic pathways. Notably, *P. wurundjeri* did not grow on SFuc-containing glycosides, indicating the absence of a sulfofucosidase capable of liberating free SFuc from glycoconjugates (data not shown). Future studies could focus on identifying and characterizing such sulfofucosidases, which would expand our understanding of how SFuc is accessed and degraded in diverse environmental contexts.

## MATERIALS AND METHODS

### Specialist chemicals

Sulfolactate and sulfolactaldehyde were synthesized as reported (50, 51). The synthesis of sulfofucose and sulfogalactonate from D-galactose, ^13^C_6_-sulfofucose from ^13^C_6_-D-galactose, and sulfotagatose from D-galacturonic acid will be reported elsewhere.

### Bacterial growth media

Growth media were prepared using M9 minimal salts media (2 mL), trace metal solution (0.1 mL), and vitamin solution (0.01 mL) and contained 10 mM SFuc as the sole carbon source, made up to a final volume of 10 mL with deionized water. M9 minimal salts media contains 0.45 M Na_2_HPO_4_, 0.11 M KH_2_PO_4_, 0.09 M NH_4_Cl, 0.04 M NaCl, 0.1 M MgSO_4_, and 0.1 M CaCl_2_. Trace metal solution contains 0.4 mM FeCl_3_, 0.08 mM CoCl_2_, 0.08 mM CuCl_2_, 0.08 mM MnCl_2_, 0.55 mM ZnCl_2_, 0.01 mM NiCl_2_, 0.05 mM Na_2_MoO_4_, and 0.5 mM H_3_BO_3_. Vitamin mixture contains 0.04 mM biotin, 0.05 mM calcium pantothenate, 0.15 mM thiamine hydrochloride, 0.36 mM p-aminobenzoic acid, 0.81 mM nicotinic acid, 1.49 mM pyridoxamine dihydrochloride, and 0.01 B12 (cyanocobalamin). Growth curves were measured using a custom-built MicrobeMeter portable spectrophotometer (52).

### Isolation of *P. wurundjeri* strain Merri

*P. wurundjeri* strain Merri was isolated by substrate-guided enrichment culturing from soil sampled at Joe’s Market Garden (34 Edna Grove, Coburg, Victoria 3058; 37.7451° S, 144.9813° E). Approximately 1 g of soil was suspended in 5 mL of sterilized M9 media (supplemented with trace metals and vitamins) containing 10 mM SFuc as the sole carbon source. The culture was incubated at 30°C for 4 d with agitation at 250 rpm. A subsample (100 µL) was transferred into fresh M9 media (supplemented with trace metals and vitamins) and grown for a further 4 days. This step was repeated five times, and after outgrowth of the final culture for 4 days, the cells were plated onto SFuc-agar plates (20 mM SFuc, 15 g/L agar) and incubated at 30°C in the dark. After 3 days, a single colony was picked and inoculated into fresh M9 media (supplemented with trace metals and vitamins) containing 10 mM SFuc and incubated at 30°C while shaking at 250 rpm. Once the culture was visibly turbid, the cells were again plated onto SFuc-agar plates, incubated at 30°C in the dark, and a single colony was picked and inoculated again into fresh vitamin-supplemented M9 media containing 10 mM SFuc. Once the culture was visibly turbid, frozen stocks were prepared by diluting to 5% DMSO and freezing at −80°C. This strain could grow on racemic DHPS (5 mM) but did not grow on SQ (5 mM), or *S/R*-SL (5 mM).

### Genome sequence, assembly, and annotation

*P. wurundjeri* strain Merri was grown in 10 ml M9 media supplemented with 10 mM SFuc for 5 days, and then, the cell pellet was harvested by centrifugation. Genomic DNA was isolated using the GenElute DNA extraction kit (Sigma) with inclusion of lysozyme and RNAase, then it was sequenced using Illumina NextSeq platform (Doherty Microbial Genomics, University of Melbourne, Australia). DNA was prepared for sequencing using the Nextera XT DNA preparation kit (Illumina) with ×150 bp paired-end chemistry and with a targeted sequencing depth of > 50× . A draft genome was assembled using Shovill v1.0.9 (https://github.com/tseemann/shovill) and annotated using PGAP as part of the NCBI Genome submission pipeline. 16S ribosomal RNA alignment was performed by extracting the 16S rRNA gene from the annotated *P. wurrundjeri* genome sequence and using this nucleotide sequence as a search query using megablast with the web version of BLASTn (https://blast.ncbi.nlm.nih.gov/Blast.cgi) against the NCBI rRNA/ITS database (15 Jun 2025 update) (30). All other search parameters were set to default values. GTDB-TK v2.1.1 with reference data version r207 was used to determine average nucleotide identity and taxonomic placement (53–55). The “classify” workflow was used with the command “gtdbtk classify_wf,” which incorporates an ANI screen using Mash, gene calling, and identification using Prodigal and HMMER, construction of a multiple sequence alignment, and classification of this alignment within a reference tree (53–55).

### ^13^C-NMR analysis of spent culture media from the growth of *P. wurundjeri* strain Merri on (^13^C_6_)-sulfofucose

M9 minimal media (supplemented with trace metals and vitamins) (4 mL) containing 10 mM (^13^C_6_)-SFuc was inoculated with *P. wurundjeri* strain Merri and grown to mid-log phase (5 days) and stationary phase (14 days) at 30°C (250 rpm). The final A_600_ was 0.3 (5 days) and 0.52 (14 days). The cells were sedimented by centrifugation at 5,000 × *g* for 3 min, and the supernatant was collected. In total, 540 µL of the culture supernatant was diluted with 60 µL D_2_O, and ^13^C-NMR spectra were acquired using a 500 MHz spectrometer. A control sample contained 10 mM (^13^C_6_)-SFuc in M9 minimal media prepared in the same way for NMR analysis.

### Analysis of sulfofucose, sulfite, and sulfate in *P. wurundjeri* culture

M9 minimal media (supplemented with trace metals and vitamins) 50 mL containing 10 mM SFuc were inoculated with *P. wurundjeri* strain Merri and grown at 30°C at 250 rpm in a 500 mL baffled conical flask. Samples were withdrawn at various time points, the cells were pelleted by centrifugation for 5 min at 5,000 × *g*, and the supernatant samples were frozen and stored at −80°C prior to analysis for reducing sugar, sulfate, or sulfite content.

### Reducing sugar assay

The reducing sugar assay was performed according to the procedure of Blakeney and Mutton (56). Alkaline diluent was prepared by the addition of sodium hydroxide (20 g, 0.50 mol) to a solution of 0.10 M trisodium citrate (50 mmol, 500 mL) and 0.02 M calcium chloride (13 mmol, 500 mL). PAHBAH working solution was prepared by dissolving 4-hydroxybenzhydrazide (PAHBAH) (0.25 g, 1.6 mmol) in alkaline diluent (50 mL). The PAHBAH working solution should be made fresh shortly before use. To determine the reducing sugar concentration, 90 µL of PAHBAH working solution and 10 µL of culture supernatant were combined in a 96-well half-area microplate. The mixture was heated at 98°C for 5 min, and the absorbance was read at 415 nm using a UV/visible spectrophotometer. Concentrations of SFuc were determined by reference to a standard curve constructed using SFuc. Analyses were performed in duplicate, and errors are reported as standard deviation.

### Turbidometric sulfate assay

The sulfate assay was performed according to the procedure of Sörbo (57). This assay uses a barium sulfate–polyethylene glycol (Ba-PEG) reagent, which contains polyethylene glycol (PEG) to stabilize BaSO_4_ crystals and a small amount of preformed BaSO_4_ seed crystals to improve the reproducibility and linearity of the assay. Ba-PEG reagent was prepared by dissolving BaCl_2_ (42 mg, 0.20 mmol) and polyethylene glycol 6000 (0.75 g) in deionized water (5.0 mL). A small amount of Na_2_SO_4_ (10 µL, 50 mM) was added to this solution, with efficient magnetic stirring to generate preformed BaSO_4_ seed crystals. The Ba-PEG reagent must be prepared fresh on the day of use. Individual sulfate assays were conducted as follows: In an Eppendorf tube, 50 µL of 0.5 M HCl was added to 50 µL culture supernatant, followed by Ba-PEG reagent (50 µL). The mixture was mixed vigorously, and then, 90 µL was transferred to a 96-well half-area microplate. The absorbance of the sample at 400 nm was measured using a UV/visible spectrophotometer. Concentrations of sulfate were determined by reference to a standard curve constructed using 0–10 mM Na_2_SO_4_. Analyses were performed in duplicate, and errors are reported as standard deviation.

### Colorimetric fuchsin sulfite assay

The fuchsin sulfite assay was performed according to the procedures of Brychkova et al. (58) and Kurmanbayeva et al. (59). This method requires three freshly prepared solutions: Reagents A, B, and C. Reagent A was prepared by adding 1 mL Schiff’s reagent to 8 mL deionized water, followed by the addition of 5 M HCl until the solution turned colorless. Reagent B was prepared by diluting formaldehyde (36% in H_2_O, 0.32 mL) in deionized water (9.68 mL) at 0°C. Reagent C was prepared by dilution of Reagent A (1 mL) in deionized water (7 mL), prior to the addition of solution Reagent B (1 mL). Individual sulfite assays were performed by adding Reagent C (100 µL) to the cell supernatant (10 µL). The sample was incubated at 25°C for 10 min and then transferred to a 96-well half-area microplate. The absorbance of the sample at 570 nm was determined by using a UV/visible spectrophotometer. Concentrations of sulfite were determined by reference to a standard curve constructed using Na_2_SO_3_. Analyses were performed in duplicate, and errors are reported as standard deviation.

### Comparative proteomics

Six independent cultures of *P. wurundjeri* strain Merri were grown in 10 mM SFuc, and another six independent cultures were grown in 10 mM glucose (both in M9 media supplemented with trace metals and vitamins). The cultures were harvested at mid-log phase (A_600_ approx 0.25), and the bacteria were collected by centrifugation at 4,000 × *g* for 10 min. The pelleted cells were washed by resuspension with fresh 10 mL ice-cold phosphate-buffered saline (PBS) and re-pelleting three times, with the supernatant discarded each time. The cells were resuspended and transferred to a 1.5 mL tube using ice-cold PBS (1 mL), and collected by centrifugation for 2 min at 10,000 × *g*, and then snap-frozen in liquid nitrogen.

Frozen whole bacterial pellets were prepared using the in-StageTip preparation approach as previously described (60). The cells were resuspended in 4% sodium deoxycholate (SDC), 100 mM Tris, pH 8.0, and boiled at 95°C with shaking for 10 min to aid solubilization. Samples were allowed to cool for 10 min and then boiled for a further 10 min before the protein concentration was determined by bicinchoninic acid assays (Thermo Fisher Scientific); 100 µg of protein equivalent for each sample was reduced/alkylated with the addition of tris-2-carboxyethylphosphine hydrochloride and iodoacetamide (final concentrations 20 mM and 60 mM, respectively), by incubating in the dark for 1 h at 45°C. Following reduction/alkylation, the samples were digested overnight with trypsin (1/33 wt/wt Solu-trypsin, Sigma) at 37°C with shaking at 1,000 rpm. Digests were then halted with the addition of isopropanol and trifluoroacetic acid (TFA) (50% and 1%, respectively), and the samples were cleaned up using home-made SDB-RPS StageTips prepared according to previously described protocols (60–62). SDB-RPS StageTips were placed in a Spin96 tip holder to enable batch-based spinning of samples and tips conditioned with 100% acetonitrile; followed by 30% methanol, 1% TFA, followed by 90% isopropanol, 1% TFA, with each wash spun through the column at 1,000 × *g* for 3 min. Acidified isopropanol/peptide mixtures were loaded onto the SDB-RPS columns then spun through, and the tips washed with 90% isopropanol and 1% TFA, followed by 1% TFA in deionized water. Peptide samples were eluted with 80% acetonitrile and 5% ammonium hydroxide and dried by vacuum centrifugation at room temperature, then were stored at −20°C.

Prepared digested proteome samples were re-suspended in Buffer A* (2% acetonitrile, 0.01% TFA) and separated using a two-column chromatography setup composed of a PepMap100 C18 20 mm by 75 µm trap and a PepMap C18 500 mm by 75 µm analytical column (Thermo Fisher Scientific). Samples were concentrated onto the trap column at 5 µL/min for 5 min with Buffer A (0.1% formic acid, 2% DMSO) and then infused into an Orbitrap Fusion Eclipse at 300 nL/minute via the analytical columns using a Dionex Ultimate 3000 UPLCs (Thermo Fisher Scientific); 125-minute analytical runs were undertaken by altering the buffer composition from 2% Buffer B (0.1% formic acid, 77.9% acetonitrile, and 2% DMSO) to 22% B over 95 min, then from 22% B to 40% B over 10 min, then from 40% B to 80% B over 5 min. The composition was held at 80% B for 5 min, and then dropped to 2% B over 2 min, then was held at 2% B for another 8 min. The Fusion Eclipse Mass Spectrometer was operated in a data-dependent mode with a single Orbitrap MS scan (375-1500 *m/z* and a resolution of 120 k) acquired every 3 s, followed by Orbitrap MS/MS higher energy collisional dissociation (HCD) scans of precursors (stepped NCE 25,30,40%, with a maximal injection time of 80 ms with the Automatic Gain Control set to 400% and the resolution to 30 k).

Identification and LFQ analysis were accomplished using MaxQuant (v1.6.17.0)(63) using the in-house generated proteome of *P. wurundjeri* strain Merri (RES21-00833.faa), allowing for the oxidation on methionine. The LFQ and “Match Between Run” options were enabled to allow comparison between samples. The resulting data files were processed using Perseus (v1.4.0.6)(64) to compare the growth conditions using Student’s *t*-tests as well as Pearson correlation analyses. For LFQ comparisons, independent replicates were grouped, and missing values were imputed based on the observed total peptide intensities with a range of 0.3σ and a downshift of 1.8σ.

### Cell-free lysate analysis

*P. wurundjeri* strain Merri cells were grown in vitamin-supplemented M9 minimal media (250 mL) containing 5 mM SFuc. Cell pellets were collected at mid-log phase by centrifugation at 4,000 × *g* for 10 min at 5°C, washed thrice with cold PBS buffer (150 mM), thrice with cold water, and frozen in liquid nitrogen. Cells were defrosted and resuspended in 100 mM Tris-HCl buffer (pH 7.0, 100 mM) and lysed by sonication with 30% powder in 3 × 30 s intervals. Samples were cooled on ice for 1 min between cycles. The samples were clarified by centrifuging at 25,000 × *g* for 10 min at 5°C, and the cell lysate was used without further treatment.

### Sulfofucose isomerase assay

Potential SFuc isomerase reactions were monitored by ^1^H NMR spectroscopy using a 500 MHz Bruker NEO system. A 700 µL sample containing 50 µL of strain Merri cell lysate, 50 units of recombinant shrimp alkaline phosphatase (Sigma), in 50 mM sodium phosphate, 150 mM NaCl (pH 7.01) was incubated for 20 min. SFuc was added to a final concentration of 10 mM, and samples were incubated for 24 h at 25°C. Reactions were quenched by heating at 80°C for 3 min, concentrated *in vacuo,* and the residue was dissolved in D_2_O for ^1^H NMR analysis.

### Sulfofucose dehydrogenase assay

In a 96-well plate, 200 µL solutions contained 1.1 mM SFuc, 6.5 mM MgCl_2_, and 0.5 mM NAD^+^ or NADP^+^ in Tris-HCl (100 mM, pH 7.5). Reactions were initiated by the addition of 20 µL of strain Merri cell lysate. Absorbance was measured at 340 nm for 15 min.

### Metabolomic analysis of *P. wurundjeri* strain Merri

Three independent replicates of *P. wurundjeri* strain Merri were grown to mid-log phase in vitamin-supplemented M9 minimal media with either 5 mM SFuc or glucose as the sole carbon source. Samples were grown to the following A_600_ values: SFuc: 1.126, 1.130, and 1.123; Glucose: 1.06, 1.01, and 1.10. Cultures were centrifuged (4,000 rpm, 30 cm rotor, 10 min, 5°C), and the cell pellets were washed three times with PBS buffer (10 mM, NaCl 150 mM, pH 7.0) and then three times with deionized water. Cells were snap-frozen with liquid nitrogen prior to extraction.

In total, 250 µL of MTBE:MeOH (3:1 vol/vol) and 125 µL of H_2_O/MeOH (3:1 vol/vol) were added to the frozen cell pellets, and the cells were lysed by sonication in a soniclean 160TD at 100% power for 10 min. Solutions were then mixed at 4 ° C for 30 min at 1,200 rpm in a ThermoMixer. Samples were centrifuged at 6,000 × *g* for 45 min, and the aqueous phase was separated and evaporated to dryness. The residues were reconstituted with 40 µL of MeOH:H_2_O (2:3 vol/vol), and the metabolome of *P. wurundjeri* strain Merri was analyzed by ion chromatography-mass spectrometry (IC-MS).

IC-MS analysis was conducted on a ThermoFisher Scientific LC ID-X Orbitrap equipped with a Dionex IonPac AS11-HC column (ThermoFisher Scientific).

A 10 µL sample aliquot was introduced onto a heated (30°C) Dionex IonPac AS11-HC analytical column (2 mm × 250 mm, 9 µm particle size) with its corresponding AG11-HC guard column (2 mm × 50 mm) using a Dionex ICS-6000 HPIC system (Thermo Scientific, USA) equipped with an integrated KOH eluent generator using a flow rate of 380 µL/min. The elution gradient consisted of 2 mM KOH held for 0.3 min, followed by linear increases to 12 mM over 13.2 min, to 20 mM over 9 min, and to 70 mM over an additional 9 min. The concentration was maintained at 70 mM for 6 min before returning to 2 mM within 0.1 min, with a subsequent re-equilibration period of 9.4 min. Sample diversion to the mass spectrometer began at the 2-min mark. An AXP-MS pump (Thermo Scientific, USA) delivered acetonitrile as make-up flow at 0.12 mL/min, which was introduced into the eluent stream via a T-split (Thermo Scientific, USA) positioned downstream of the conductivity detector and as proximal to the ionization source as feasible.

MS acquisition was performed using a Thermo Scientific ID-X system operating at 240,000 resolution (referenced at m/z 200). The chromatographic eluent was introduced through a heated electrospray ionization (HESI) source maintained at 225°C with optimized gas parameters (sheath: 58 arbitrary units, auxiliary: 14 arbitrary units, sweep: 0.5 arbitrary units). Metabolites were ionized in negative mode using a 3000 V spray voltage with mild trapping enabled and RunStartEasyIC calibration with selected ion monitoring in negative ionization mode to monitor for SFuc [M-H]^-^ *m/z* 243, SGalL [M-H]^-^ *m/z* 241, SGal [M-H]^-^ *m/z* 249, KDSGal [M-H]^-^ *m/z* 241, SLA [M-H]^-^ *m/z* 153, and SL [M-H]^-^ *m/z* 169. Ions monitored were fragmented by CID (0–30 arbitrary units) or HCD (0–45 arbitrary units). Additional desolvation occurred in the transfer tube heated to 300°C. Data were collected across a mass range of 100–1,000 m/z with default maximum injection time and automatic gain control (AGC) target settings, and an RF lens value of 35%. Fragmentation spectra were acquired using data-dependent acquisition (ddMSn) with higher energy collisional dissociation (HCD) at assisted stepped collision energies (15%, 30%, and 45%) at 60,000 resolution using a 1 Dalton isolation window. A second ddMSn event employed collision-induced dissociation (CID) at 30% collision energy with a 1.6 Dalton isolation window at the same resolution. All scans used [M-H]^-^ *m/z* 243, 241, 259, 169, and 153 inclusion lists.

### Cloning, protein expression, and purification of SfcD, SfcE, SfcF, SfcH, and MDP0926266

The codon-harmonized protein-coding regions of SfcD, SfcE, SfcF, SfcH, and MDP0926266 genes from *P. wurundjeri* strain Merri (MDP0926279, MDP0926278, MDP092627, MDP0926275, and MDP0926266, respectively) were synthesized and cloned between *Nco*I and *Xho*I in pET-28a with an N-terminal hexahistidine tag, followed by the TEV protease recognition site. For protein production, plasmids encoding SfcE, SfcF, SfcG, or MDP0926266 were transformed into *E. coli* BL21(DE3) and expressed. The cells were grown in lysogeny broth with 50 mg/mL kanamycin at 37°C, and when they reached an A_600_ of 0.6–0.8, they were induced with 0.4 mM isopropyl 1-thio-β-D-galactopyranoside (IPTG). After incubation at 18°C for 16 h, cells were harvested by centrifugation at 6,000 × *g*. Due to the low solubility of the SfcD protein, the plasmid encoding SfcD was expressed in ArcticExpress (DE3) (Agilent). The transformed cells were grown in LB media at 30°C, and when they reached an A_600_ of 0.6–0.8, they were induced with 0.1 mM IPTG and incubated at 11°C for 24 h, then harvested as above. The cell pellets were resuspended in buffer A (20 mM Tris

HCl, 500 mM NaCl, 25 mM imidazole, pH 7.5), disrupted using a sonicator (Omni Sonic Ruptor 400), and clarified by centrifugation at 20,000 × *g*. The soluble fraction was applied to Ni Sepharose 6 Fast Flow (Cytiva) resin, and the bound protein was eluted with 300 mM imidazole in buffer A. Pooled fractions were concentrated and buffer exchanged into 20 mM Tris

HCl, 200 mM NaCl, pH 7.5.

### Analysis of the products of reactions catalyzed by SfcD, SfcE, SfcF, and SfcH

SFuc dehydrogenase (SfcH): A 0.5 mL solution contained SFuc (2 mM), and either NAD^+^ (3 mM) or NADP^+^ (3 mM) in Tris-HCl buffer (50 mM, pH 8.0). SfcG (1.67 µM) was added to initiate the reaction, and the solution was incubated at 25°C for 24 h, then halted by heating at 80°C for 3 min. Samples were frozen until LC-MS analysis.

SGalL lactonase (SfcD): A 0.3 mL solution contained 100 µL of the final SfcG solution from above, CaCl₂ (0.5 mM), and ZnCl₂ (0.5 mM) in Tris-HCl buffer (50 mM, pH 8.0). The reaction was initiated by adding SfcG (2.67 µM), and the solution was incubated at 25°C for 24 h, then halted by heating at 80°C for 3 min. Samples were frozen until LC-MS analysis.

SGal dehydratase (SfcF): A 0.5 mL solution contained chemically synthesized SGal (2 mM) and MgCl₂ (1 mM) in Tris-HCl buffer (50 mM, pH 8.0). The reaction was initiated by adding SfcF (1.11 µM) and incubated at 25°C for 24 h, then halted by heating at 80°C for 3 min. Samples were frozen until LC-MS analysis.

KDSGal aldolase (SfcE): A 0.5 mL solution contained 100 µL of the final SfcF solution from above, and either MgCl₂ (1 mM), MnCl₂ (1 mM), or ZnCl₂ (1 mM) in Tris-HCl buffer (50 mM, pH 8.0). The reaction was initiated by adding SfcE (1.68 µM) and incubated at 25°C for 24 h, then halted by heating at 80°C for 3 min. Samples were frozen until LC-MS analysis.

LC-MS analysis: In total, 20 µL of each sample was diluted with 60 µL of 1:1 ACN/H_2_O and used for LC-MS analysis. An Agilent 6490 QQQ mass spectrometer equipped with an Agilent LC module was used for HPLC–MS analysis. A SeQuant ZIC-HILIC 3.5 µm 100 Å 150 × 2.1 mm column was used at room temperature. HPLC used solvent A, 10 mM NH_4_OAc in 10% acetonitrile; solvent B: acetonitrile. The elution gradients were from 90% B to 40% B over 50 min; then 40% B for 2 min; back to 90% B over 1 min with a flow rate of 0.50 ml min^−1^ and an injection volume of 1 µL. The mass spectrometer was operated in negative ionization mode. Prior to analysis, the sensitivity for each multiple reaction monitoring (MRM)-MS^2^ transition was optimized for each analyte.

Retention times and MS^2^ fragmentation patterns of the authentic reference standards were as follows:

For SFuc: Retention time: 25.3 and 25.8 min. ESI–MS/MS *m/z* of [M-H]ˉ 243, product ions 283, 153, 123, and 81.

For SGalL: Retention time: 13.1 min. ESI–MS/MS *m/z* of [M-H]ˉ 241, product ions 197, 179, 161, and 81.

For SGal: Retention time: 27.0 min. ESI–MS/MS *m/z* of [M-H]ˉ 259, product ions 241, 213, 197, 183, 179, 161, and 81.

For KDSGal: Retention time: 22.6 min. ESI–MS/MS *m/z* of [M-H]ˉ 241, product ions 223, 205, 179, 161, and 81.

For SLA: Retention time: 2.1 min. ESI–MS/MS *m/z* of [M-H]ˉ 153, product ions 81 and 71.

IC-MS analysis of pyruvate: Following the incubation of KDSGal aldolase reaction mixtures, the enzyme was precipitated by adding CHCl₃ (300 µL), then centrifuged at 1,400 rpm (using a 15 cm rotor) for 10 min. The aqueous layer was collected and diluted to a final pyruvate concentration of 10 µM for IC-MS analysis in a manner analogous to that previously described with selected ion monitoring in negative ionization mode to monitor for pyruvate [M-H]^-^ *m/z* 87. All scans used [M-H]^-^ *m/z* 87 inclusion lists.

### Investigation of the activity of MDP0926266

Solutions (150 µL) were prepared containing 1.0 mM racemic SLA, 1.5 mM NAD^+^ or NADP^+^ in 100 mM Tris-HCl (pH 7.5), with or without 0.5 mM ZnCl_2_, FeCl_2_, or FeCl_3_. Reactions were initiated by the addition of MDP0926266 to a final concentration of 30 nM. Absorbance at 340 nm was monitored over 15 min. No enzymatic activity was detected under any of the tested conditions.

### Bioinformatic analysis

The protein sequence of SfcF (UniProt accession code: Q5LUS2) from the *P. wurundjeri* strain Merri was used as a query for BLAST search in the Enzyme Function Initiative-Enzyme Similarity Tool (EFI-EST webtool, ENA database, version June 2022; UniProt release 2022_02 and InterPro 89) (39, 40). This search retrieved 1001 sequences with ≥62.2% identity. Sequence similarity networks (SSNs) were generated at different alignment scores (AS = 120, 140, 160, 180, and 200) and visualized in Cytoscape (65) 3.10.3 using the yFiles Organic Layout, with nodes representing individual sequences.

Genome neighborhoods were analyzed using the Enzyme Function Initiative-Genome Neighborhood Tool (EFI-GNT), with nodes color-coded according to colocation with SfcD, SfcE, SfcF, and SfcH homologs. Analysis at AS = 160 identified two clusters containing seven sequences with SfcF and at least one homolog of SfcD, SfcE, or SfcH.

Further analysis of the SSN at AS = 160 using EFI-GNT retrieved neighboring genes within a ± 10 open reading frame window.

## Data Availability

The assembled genome has been deposited at the NCBI (GenBank accession: Bioproject PRJNA993841), release date Aug 7, 2023. The mass spectrometry proteomics data has been deposited in the Proteome Xchange Consortium(66) via the PRIDE partner repository (https://proteomecentral.proteomexchange.org/cgi/GetDataset) with the data set identifier: PXD043488.

## SUPPLEMENTAL MATERIAL

Supplemental Figures mbio.01840-25-s0001.docx Figures S1-S8.

Proteomics Data mbio.01840-25-s0002.xlsx Table of proteomics data.

Supplemental Information mbio.01840-25-s0003.docx Gene sequences of proteins heterologously expressed in *E. coli*.

Supplemental Tables mbio.01840-25-s0004.docx Tables S1–S6.

ASM does not own the copyrights to Supplemental Material that may be linked to, or accessed through, an article. The authors have granted ASM a non-exclusive, world-wide license to publish the Supplemental Material files. Please contact the corresponding author directly for reuse.
