# Supplementary material for: Tandem sulfofucolytic-sulfolactate sulfolyase pathway for catabolism of the rare sulfosugar sulfofucose
Source: mBio. 2025 Aug 18;16(9):e01840-25. doi: 10.1128/mbio.01840-25 (PMC12421959; doi:10.1128/mbio.01840-25)
Supplement: Supplemental Figures — Figures S1-S8. [file mbio.01840-25-s0001.docx]

SUPPLEMENTAL FIGURES


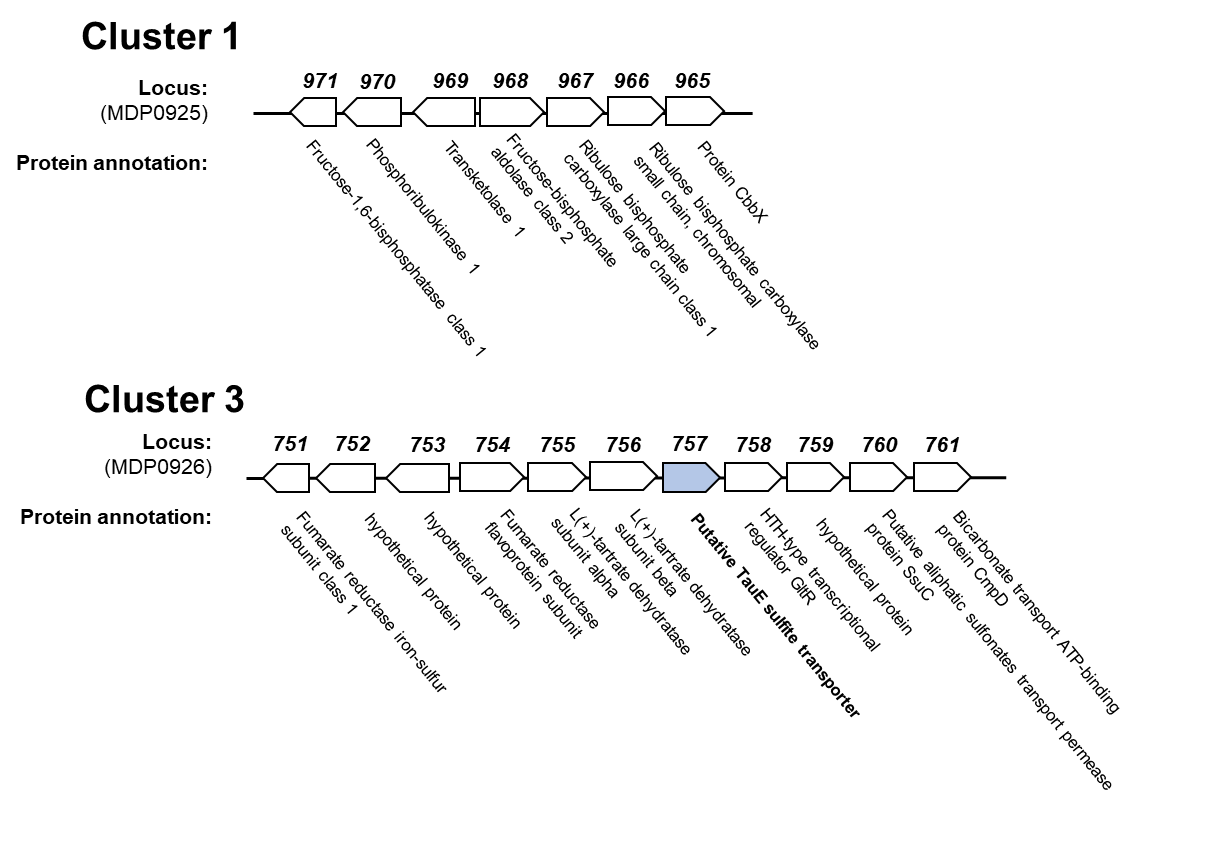


**Figure S1.** Gene clusters 1 and 3 identified by comparative proteomics. Annotations were added automatically during assembly and annotation, except for annotation for TauE added manually.
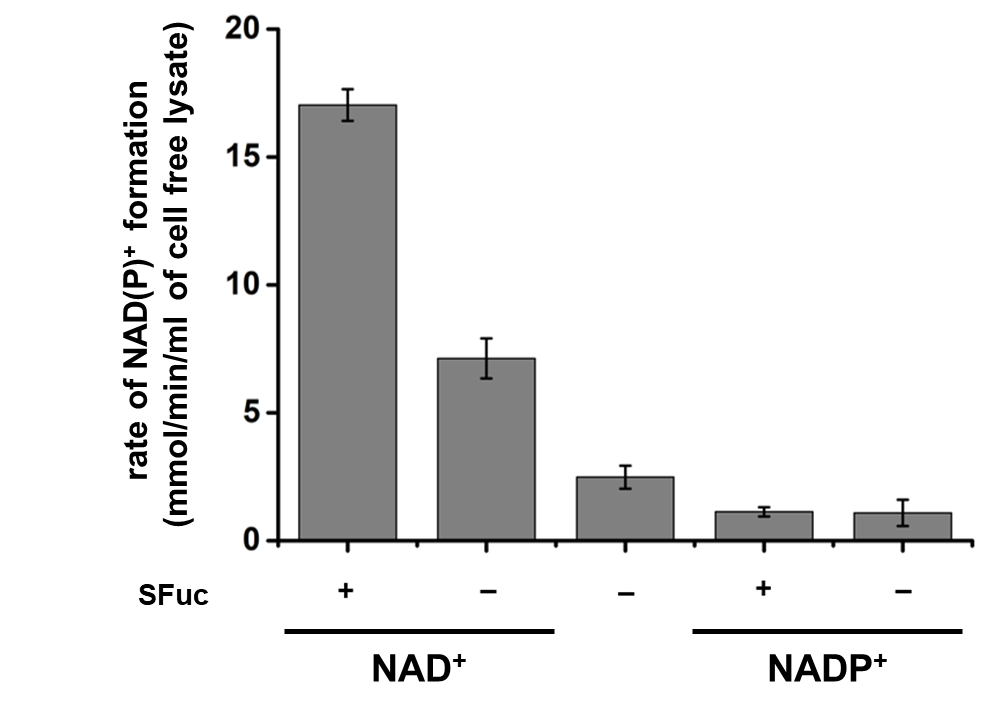


**Figure S2.** NAD^+^-dependent sulfofucose dehydrogenase activity of cell free lysate of *P. wurundjeri* strain Merri. Reactions contained 1.1 mM SFuc, 6.5 mM MgCl_2_, and 0.5 mM NAD^+^ or NADP^+^ in Tris-HCl (100 mM, pH 7.5). Reactions were initiated by the addition of 20 μL of strain Merri cell lysate and absorbance was measured at 340 nm.


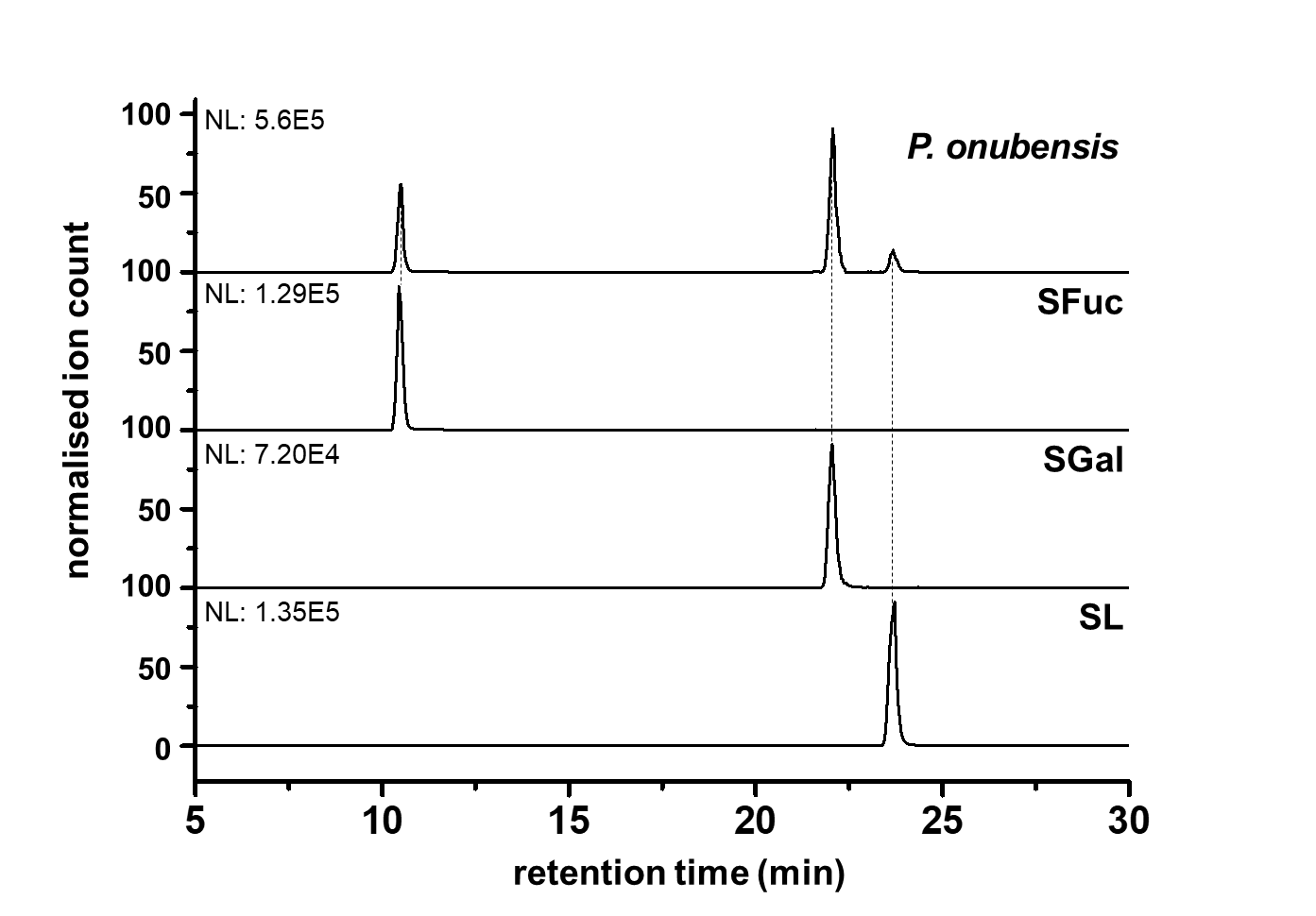


**Figure S3. Ion chromatography-mass spectrometry analysis of *P. wurundjeri*** **strain Merri grown on SFuc shows SGal and SL as metabolites.** Extracted ion chromatographs of metabolites from *P. wurundjeri* (top), and chemically synthesised SFuc, SGal and SL.


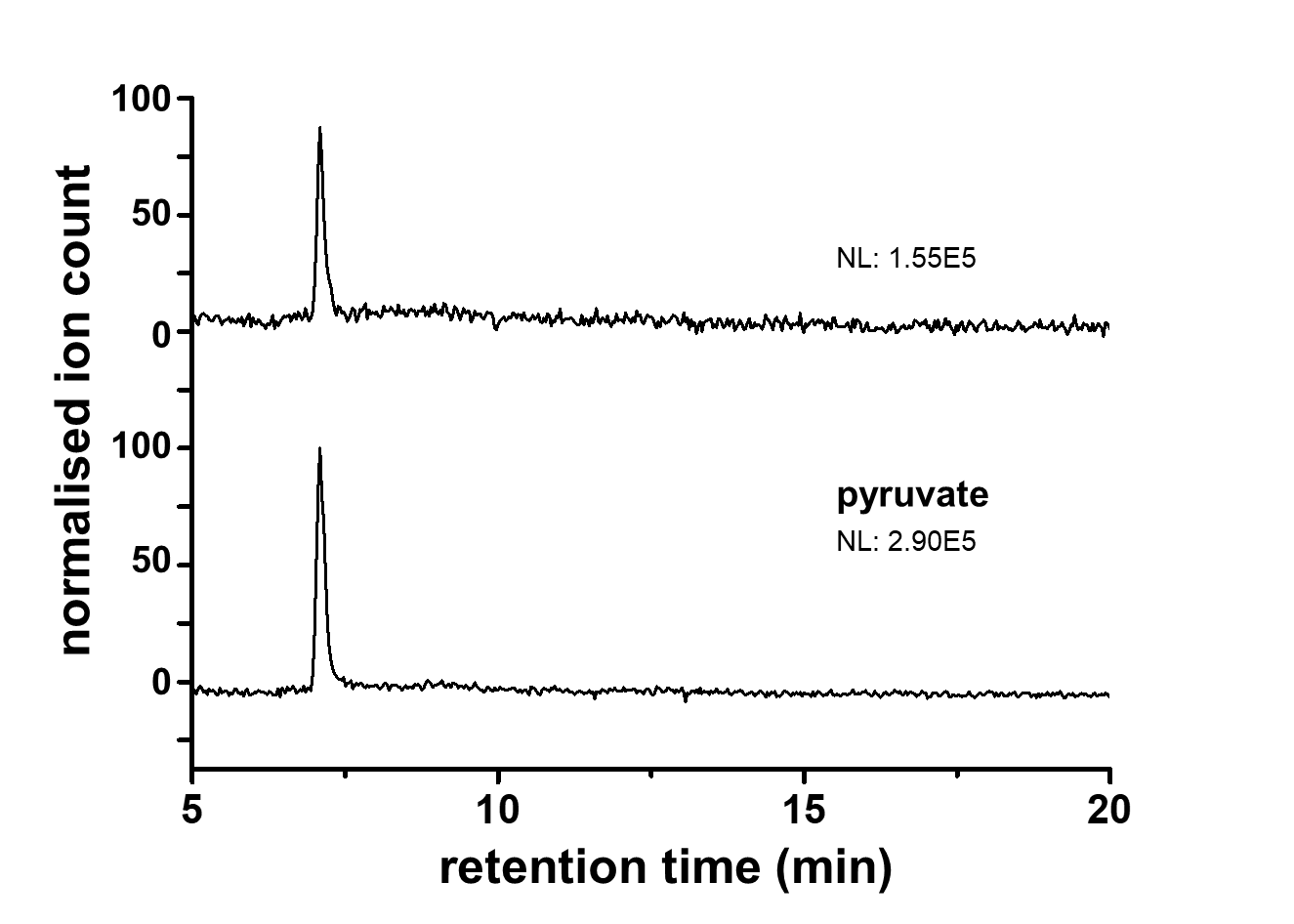


**Figure S4.** **Pyruvate is generated by KDSGal aldolase (SfcE).** Targeted SIM IC-MS extracted ion chromatographs monitoring for *m/z* 87 (pyruvate) showing (top) reaction mixture containing KDSGal, MgCl_2_ and SfcE and (bottom) a pyruvate standard.


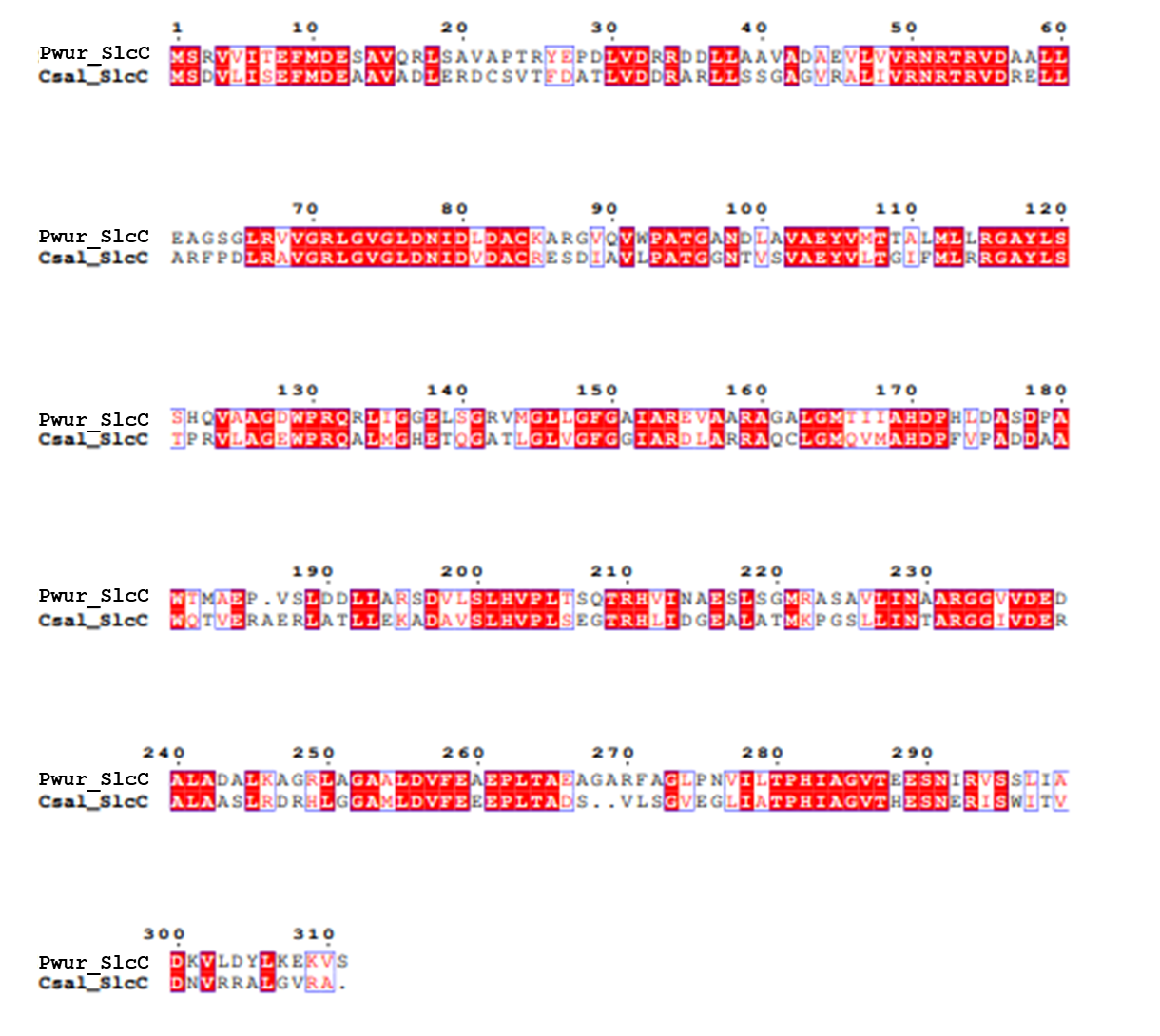


**Figure S5.** Sequence alignment of proposed SlcC protein from *Paracoccus wurundjeri* strain Merri and biochemically validated SlcC from *Chromohalobacter salexigens*.


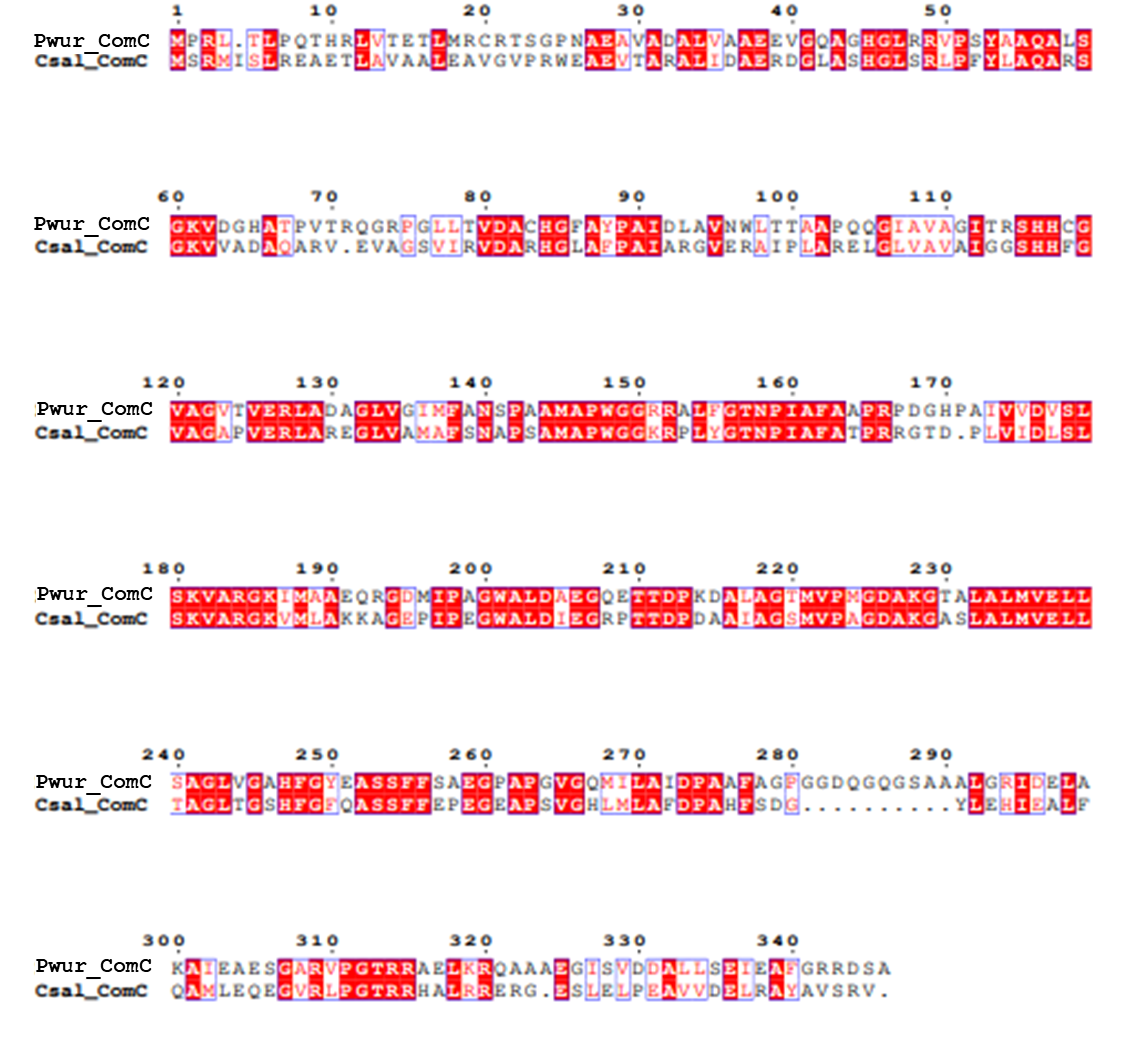


**Figure S6.** Sequence alignment of proposed ComC protein from *Paracoccus wurundjeri* strain Merri and biochemically validated ComC from *Chromohalobacter salexigens*.

**
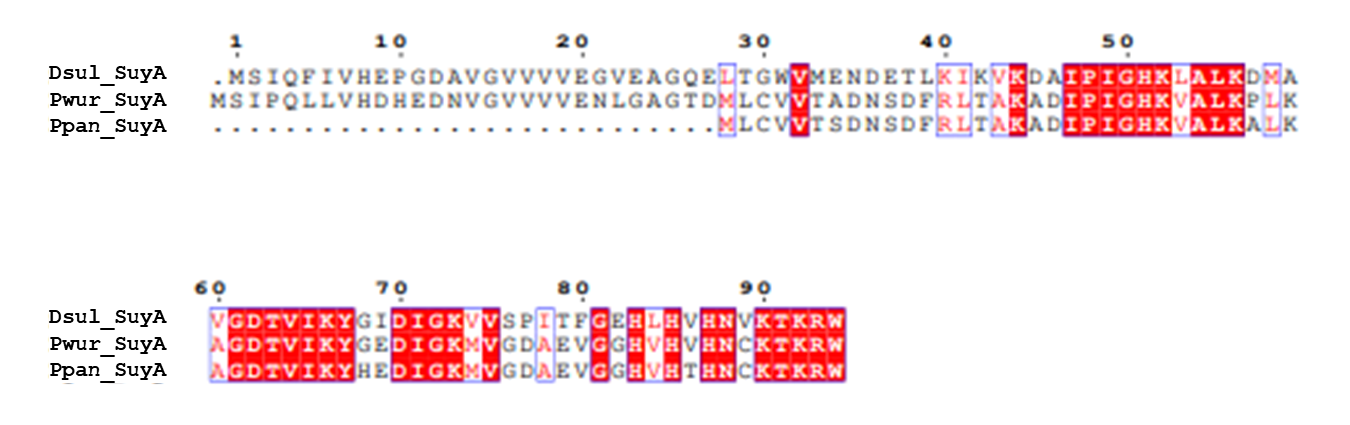
**

**Figure S7.** Sequence alignment of proposed SuyA protein from *Paracoccus wurundjeri* strain Merri, and biochemically validated SuyA from *Paracoccus pantotrophus* NKNCYSA and *Desulfovibrio sp. strain* DF1.

**
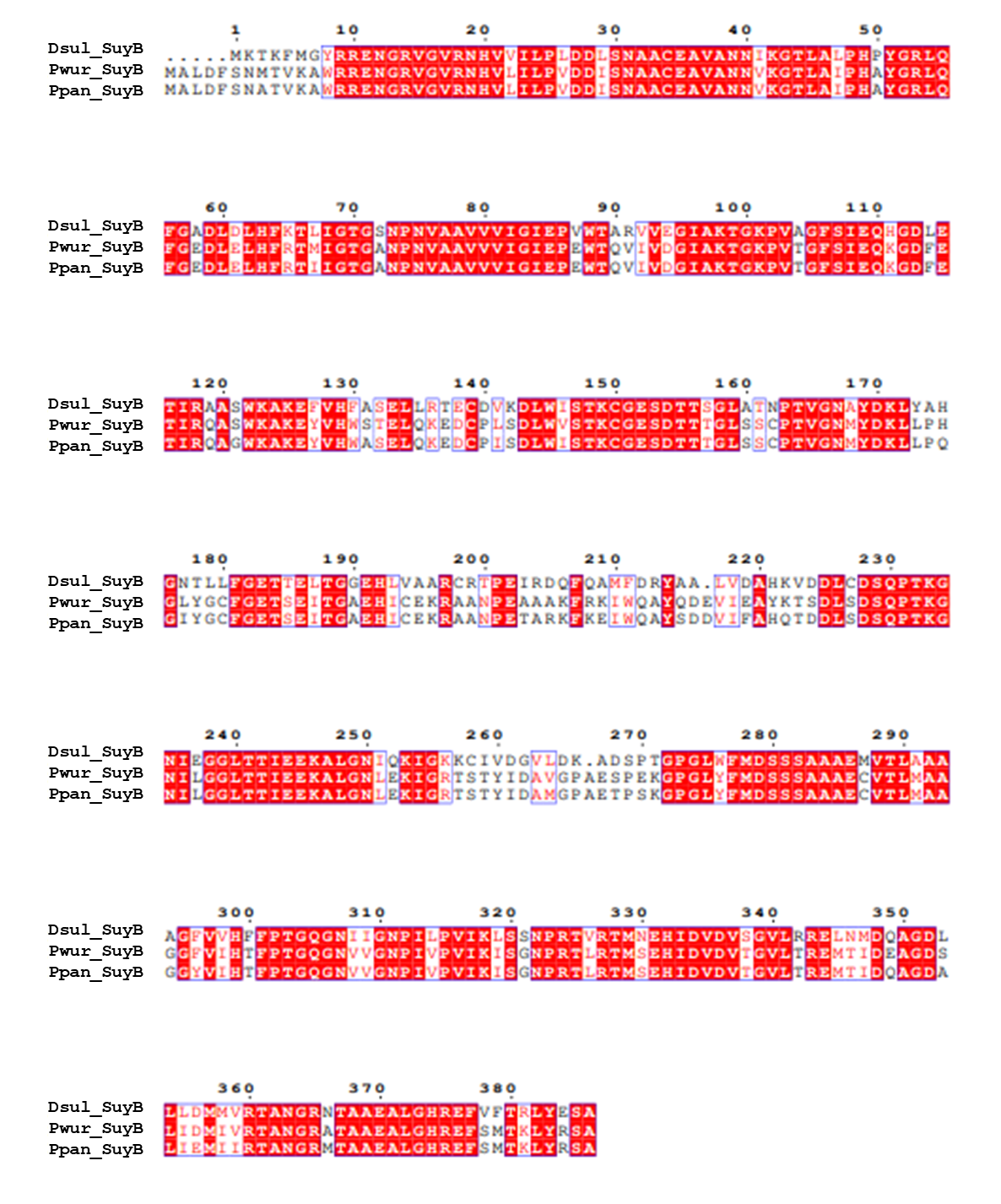
**

**Figure S8.** Sequence alignment of proposed SuyB protein from *Paracoccus wurundjeri* strain Merri, and biochemically validated SuyA from *Paracoccus pantotrophus* NKNCYSA and *Desulfovibrio sp. strain* DF1.
