# Supplementary material for: Tandem sulfofucolytic-sulfolactate sulfolyase pathway for catabolism of the rare sulfosugar sulfofucose
Source: mBio. 2025 Aug 18;16(9):e01840-25. doi: 10.1128/mbio.01840-25 (PMC12421959; doi:10.1128/mbio.01840-25)
Supplement: Supplemental Information — Gene sequences of proteins heterologously expressed in E. coli. [file mbio.01840-25-s0003.docx]

SUPPLEMENTAL INFORMATION – GENE SEQUENCES

**Codon harmonised amino acid sequences of expressed *P. wurundjeri* SfcD, SfcE, SfcF, SfcH, and MDP0926266**

> SfcD (lactonase)_codon harmonised

ATGCCTCGGGTCATTGACATTTGGCAAGTGGAGCGCCCTGAGCTCCCTCGTTCGCTCCTGGGTGAGACGGTGCGCTGGCACGCTCGATCAGGCCGTGTTATATACGCTGACATAATGGGCCGCAAGTTACTGGCATACGACCCATTGACTGGACAACAAGACGACTGGAGTTTTGAAGCACCAGTTGGTGGTTGGTTCCCTACTATTCGTGACGAGATAATTCTGGCTGTGGGCCAAAATCTTGATTGGTTTGATATGAAGACCGGGTCACGTCGTCCACTGGCCAGTCTCGTTGGTGAAGACGCAGACATACGATTTAACGATGGCAGATGCGATCCCGCAGGTCGCTTGTGGATAAGCACCATGTCCATGTCTGGCAACCCACCGCCGCCTCGTGGAAGACTTTTCCGCTTGGATCCACCAGGTATTCTTACCCCTGTAATTGATGGATTACGTATACCAAACACATTAGCGTGGTCACCTGACGGCAATAGTATGTACTTTGCTGACTCTCTTACTCGGGAGACAGGGCGTTATGGTTACGACCCTCAGAATGGGGAACTGGGAACGCGCTCTGTGCTCTTTAGATTCAGTGACGCCGTCAGTGGTATACCAGACGGCGCATGTGTAGACGAAGAGGGTGGTATATGGATCGCAGTCCCACGTGGGTCTCGTGTAGAGCGTCGTATGCCAGATGGAAGCCTTGATACCGTCATACTTCTTCCTGCAGAGCGTCCCACGATGTGCGCATTCGGTGGGGCAGATCGAGACATTTTGTTTATTACATCCCAAAGCTTGTTTCTTAGCGAGGAAGAAAGACGCTTTCGCGTAAACGATGGGGCTTTCTTTGCGGTTAGACCGGGTGTTACTGGGCTCCCCGAGGCACAGTTTGATCCTGATACAATTCGGAATTAA

> SfcE (aldolase)_codon harmonised

ATGACTACTAGAAATGACTTTGCTAACCCCTTTCGTCGCGCACTCGCTGGCAAGAAAGTTTTAACGGGAATATGGAGTATGTTAAACTCTACGAACGCCATTGAAGGACTGGGATGGGCTGGGTTTGATTGGCTCGTTATTGACGCGGAGCACTCACCTGTGTCATTGCACGATGCTATGGCCCACTTACGGGCTTTGGGTGCAACCCCCACCATACCCGTTGTCCGCCTTCCGTGGAATGACAATGTACTCATTAAACACTACTTGGACATAGGAGCTCAAACAATAATGTTGCCCCTTATTCAAAACGCGCGTGAGGCTCAAGCAGCAGTACGTGCAATGCGGTATCCCCCGGGTGGTATGCGGGGCTTTGCAGCAATGCACAGAGCATCACGATATGGTCACATACCACAGTACGTCGAGAGAGCGGAAGAGACTTTATTTATGATTGTCCAAGTCGAAACTGTAGAAGCCCTCGACCGCTTAGATGAAATTGCCAGCGTTGAGGGAGTTGATGCTGTCTTCTTCGGTCCGGGAGACTTAAGTGCGTCGATGGGCCTGTTAGGGAAACCAGGCCAAACTGAGGTATTTGATCGTATTGTGGAAGCCTCAAAGACAGTTCAACGACTGGGAAAATCGACTGGAGTGTTGGCACCGTCCATTGGGCATGCAAAGTCTTACTGTTCGGCAGGAGTCAACTTTGTTTCTGTCGCTACGGACTGTGCATTGCTCTTCCGCAATGCGGATGCCTTAGCAGCAGACTTTGCTGCTTTTACAGCAGCAGCCACCTGA

> SfcF (dehydratase)_codon harmonised

ATGGATCGCATTGCAGAGATTACTAGTTTTACAGTACCACCACGTTGGATCTTTGTGCGCGTTCGGACGGCAGAGGGATTGACAGGCTGGGGTGAAGCGATTATTCCTAAACGTCGGAACGCAGTGATAGGGGCAATACGAGACTTGACTCAAGTGGTTTTAGGAATGGACCCGGCTCGTATTGAAGACATTGCCAGTAGCCTGAGAAAAGGTTCATTCTTTCGCAATGGACCTATTCTCGGCACTGCGATCGCTGCCGTGGAGATCGCTCTGTGGGACATTAAAGGCCAAAGAGCTGGCCTGCCTGTGTTTGAATTCCTTGGCGGTCGTGTCAGAGACAACATAAGAAGCTACACGTGGATAGGTGGGGATTCTCCAGCTAACGTTGTATCTCATGCCAAGGAGAGAGTTGAGCAAGGATTTGACGCTGTGAAAATGAATGCCACACCGGCGGTTGCACACTTAGAGTGGCGTGAAGCCACTGAGAATTTAGTACAGCGAATGGGGTCTCTTCGTGACGCTTTTGGTGGTAGCATAGATATCGCCCTTGACTTTCACGGTCGTGTGCCTAGAAGTGTTCTGAAGCAAATGGTAAAAGAGATAGAACCATTTGACCCATTGTGGATTGAGGAACCCTTTCTTCCGGAGCACGTTGGTGCTGACGAATTAATGGCCCGAATTTGTCCACACATACCAATAGCGACGGGAGAGAGATTGCTTCACCGCTGGGACTTTCAAAGACTGTTAGAGAGAGGTGGGGTTGACGTTATACAACCGGATATCTCGATAACCGGTCTGTTTGAAATGGAGAAAATTGCAAGACTTGCAGAAATCTATGATGTGGGTGTGGCACCTCACTGCCCTAACGGGCCAATAAGCTTGGCGGCAAGTCTCCAAGTCGACTTTTGTTGTGCGAACACCGTTATCCAAGAACAGTCTCTGGGCTTGCATTATAACCAAGGGTACGCAGGGTTGCCACCAGCTGATATTCTTGACTACATAGGCGACCCGGACGTTCTCACAACTCGCAATGGGCGTTTTGATTGCCCGAGTGCACCAGGGCTTGGTTTAATATTGAAGGGTGACACCATTGAAGCTGCCCACACTGATTGGAGTTTACCGGACCCTGATTGGCGACACAGAGATGGTGTATACGCTGAGTGGTAA

> SfcH (dehydrogenase)_codon harmonised

ATGACAGATATCATAACTGAGCCCGGCGTTCTTGTCACCGGTGCTGCCGGTGGTATAGGTCGTGCTGTTGCAGGTTTATTTGCTGATCAGGGGCATGCTGTAACACTGACGGACAGAGACTCGGCAGGTCTTGAGGATATAGGCGGACGGTTGCGTGATCGCGGGTGCAAGGTAGATATGATTGCTCAGGATCTTGCCGATCCCGACGCACCTGCCATGTTAGTCGCCTGCACCGTCGAGAAATGGGGCGGTATTGGAGTTCTTGTGAACAATGCTGCGCATCATGGTAAACGACAGAGTGTGCTGTCGAGCGAACCAGACGAATGGCGCCAGGTTTTCGAGGTGAATGTGATAGCCGCAGCAGCGCTGGCACGGTATGCAGCTCTCGACATGGCACGTCGCAAAACGGGTGCGATCATCAATGTGGGGTCCATACAACAGGCATTGCCGGTTAGTTCCTATGCCGCATATGTCGCAAGCAAGGGTGCAGTAGCCGGGATGACTCGGGCACTTGCCGTGGAATTGGGTCTGCTGGGCATAAGAGTCAACAGTGTCAGCCCTGGTGTGATCGGCACGGAGAATTTCCGCCGTGAATTGGAAACCCGTCACGATGGCGCTTCAGCTATCAGTTATCCGTCTTTATTGGGACGCCTTGGAACGCCTGAGGATGTGGCTCATGTAATCGCGTTTCTCGCCGGTCCCCATTCTTCTCATGTAACTGGTGCAGATTACGTGGTTGATGGCGGACGTGGGATCTCACGTCGCACCGACCCTTTCCATGCCGAAATCACACATCCAGTGAGTGGTAAATAG

> MDP0926266 (Putative threonine dehydrogease tested for SLADH activity)_codon harmonised

ATGATCCCTAATGAGAAAACCATGCAAGCTGCAGACTTCTTGGGAGAGGATCGTATTGAAATAGTATCACGACCACTTCCAGAGCCTGCAGAGGGCGAGGTGTTGCTCAGAGTGGCCGCCAACGCGCTCTGTGGTTCTGACTTAAAGCTCTGGCACGCTGGGGCCCAACATATAGCTGGTCATGAAATTGCTGGTTGGGTCCAACAGCCAGGCCACCCCTTGAATGGACAACTCTGTGCAGTATACATACCCTTACACTGTGGGGAGTGTGCTGTATGCCTCAGAGGGGACACACAGTCTTGTATAACAGTTTCAAGCTTAATTGGTTGGAATAGAGACGGTGGGTACGCACAGTACTTGACGGTTCCCGAGAATTGTTTACTCCCCGTACCCGGAGACATTGACGCTGCATTAGCTCCTTTGCTCTTAGACACAATAGGGACCTCAGCACACGCACTTCGAGAAGCGTCTCGTCACCTGGCTACTGATGCACCAAGTGTGTTAGTAACTGGTGCTGGCCCCGTAGGCTTGGGTGTAGTACTTGCGGCGGCCGCCTTAGGTCACGCTCAAGTTGACGTTGCTGAACCCAACCCGGCACGGGCGGCTATAGCTCGCGAGTTTGGGGCAAACATTGTTCCTGTAGGATCACACGATAGAAGATACGACCTCATAGTTGAGTGCTCTGGTAACCACGCTGCTCGGGACCTTGCGATACACTTGGTGTTGCCGAAGGGTGTAATTGTCCTGGTTGGGGAGAATGCAGCCCCCTGGTCAGTGACAGAGGATAAAGTTTTCCGACGTAAAGACTTTGCATTACTCAGAACGTTTTACTTTCCACGTGACGACTTTGCGGCAAACGTTGAGCTTTTAAGAGCTAATCGTGAAAAGTACGCAAGACTTGTTGACGATGCTTTCCCTATTGCTGAGTTGCCTCAAAAGTTTGCAGACTTTGCTGAGGGTAAGAGTATTAAGCCGATATTAAGCTTTATTGGCGAACAGTAA
